# Defining the genotypic and phenotypic spectrum of X-linked *MSL3*-related disorder

**DOI:** 10.1038/s41436-020-00993-y

**Published:** 2020-11-11

**Authors:** Theresa Brunet, Kirsty McWalter, Katharina Mayerhanser, Grace M. Anbouba, Amy Armstrong-Javors, Ingrid Bader, Evan Baugh, Amber Begtrup, Caleb P. Bupp, Bert L. Callewaert, Anna Cereda, Margot A. Cousin, Juan C. Del Rey Jimenez, Laurie Demmer, Nikita R. Dsouza, Nicole Fleischer, Ralitza H. Gavrilova, Sumedha Ghate, Elisabeth Graf, Andrew Green, Sarah R. Green, Maria Iascone, Ameni Kdissa, Dirk Klee, Eric W. Klee, Emily Lancaster, Kristin Lindstrom, Johannes A. Mayr, Meriel McEntagart, Naomi J. L. Meeks, Dana Mittag, Harrison Moore, Anne K. Olsen, Damara Ortiz, Gretchen Parsons, Loren D. M. Pena, Richard E. Person, Sumit Punj, Gonzalo Alonso Ramos-Rivera, Maria J. Guillen Sacoto, G. Bradley Schaefer, Rhonda E. Schnur, Tiana M. Scott, Daryl A. Scott, Carolyn R. Serbinski, Vandana Shashi, Victoria M. Siu, Barbro Fossøy Stadheim, Jennifer A. Sullivan, Jana Švantnerová, Lea Velsher, David S. Wargowski, Ingrid M. Wentzensen, Dagmar Wieczorek, Juliane Winkelmann, Patrick Yap, Michael Zech, Michael T. Zimmermann, Thomas Meitinger, Felix Distelmaier, Matias Wagner

**Affiliations:** 1 Institute of Human Genetics, Technical University Munich, Munich, Germany; 2 GeneDx, Inc., Gaithersburg, MD USA; 3 Division of Genetics and Metabolism, Department of Pediatrics, University of Wisconsin School of Medicine and Public Health, Madison, WI USA; 4 Department of Pediatric Neurology, Massachusetts General Hospital, Boston, MA USA; 5 Department of Clinical Genetics, University Children’s Hospital, Paracelsus Medical University, Salzburg, Austria; 6 Institute for Genomic Medicine, Columbia University, New York, NY USA; 7 Medical Genetics, Spectrum Health and Helen DeVos Children’s Hospital, Grand Rapids, MI USA; 8 Department of Pediatrics and Human Development, College of Human Medicine, Michigan State University, Grand Rapids, MI USA; 9 Center for Medical Genetics, Ghent University Hospital, Ghent, Belgium; 10 Department of Biomolecular Medicine, Ghent University, Ghent, Belgium; 11 Department of Pediatrics, ASST Papa Giovanni XXIII, Bergamo, Italy; 12 Center for Individualized Medicine, Mayo Clinic, Rochester, MN USA; 13 Department of Health Sciences Research, Mayo Clinic, Rochester, MN USA; 14 St George’s Genomics Service, St George’s University Hospitals NHS FT, London, UK; 15 Medical Genetics, Atrium Health Levine Children’s Hospital, Charlotte, NC USA; 16 Bioinformatics Research and Development Laboratory, Genomics Sciences and Precision Medicine Center, Medical College of Wisconsin, Milwaukee, WI USA; 17 FDNA Inc., Boston, MA USA; 18 Department of Clinical Genomics, Mayo Clinic, Rochester, MN USA; 19 Department of Neurology, Mayo Clinic, Rochester, MN USA; 20 St Vincent Hospital Medical Genetics Clinic, Green Bay, WI USA; 21 Institute of Human Genetics, Helmholtz Zentrum München, Neuherberg, Germany; 22 Department of Clinical Genetics, Children’s Health Ireland at Crumlin, Dublin, Ireland; 23 University of Arkansas for Medical Sciences, Arkansas Children’s Hospital, Springdale, AR USA; 24 Laboratorio di Genetica Medica, ASST Papa Giovanni XXIII, Bergamo, Italy; 25 CENTOGENE AG, Rostock, Germany; 26 Department of Diagnostic and Interventional Radiology, Medical Faculty, Heinrich Heine University Düsseldorf, Düsseldorf, Germany; 27 UPMC Children’s Hospital of Pittsburgh, University of Pittsburgh School of Medicine, Pittsburgh, PA USA; 28 Division of Genetics and Metabolism, Phoenix Children’s Hospital, Phoenix, AZ USA; 29 Department of Pediatrics, Salzburger Landeskliniken and Paracelsus Medical University, Salzburg, Austria; 30 Medical Genetics, St George’s University Hospitals NHS FT, London, UK; 31 Department of Pediatrics, Section of Genetics, University of Colorado Anschutz Medical Campus, Aurora, CO USA; 32 INTEGRIS Pediatric Specialties/Medical Genetics, Oklahoma City, OK USA; 33 Department of Pediatric, Soerlandet Sykehus Kristiansand, Kristiansand, Norway; 34 Division of Human Genetics, Cincinnati Children’s Hospital Medical Center, Cincinnati, OH USA; 35 Department of Pediatrics, University of Cincinnati College of Medicine, Cincinnati, OH USA; 36 Department of Pediatric Neurology, National Institute of Children’s Diseases, Bratislava, Slovakia; 37 Texas Children’s Hospital, Houston, TX USA; 38 Department of Microbiology and Molecular Biology, College of Life Sciences, Brigham Young University, Provo, UT USA; 39 Department of Molecular and Human Genetics, Baylor College of Medicine, Houston, TX USA; 40 Department of Molecular Physiology and Biophysics, Baylor College of Medicine, Houston, TX USA; 41 Department of Pediatrics, Division of Medical Genetics, Duke University Medical Center, Durham, NC USA; 42 Department of Pediatrics, Western University, London, ON Canada; 43 Department of Clinical Genetics, Oslo University Hospital, Oslo, Norway; 44 Second Department of Neurology, Faculty of Medicine, Comenius University, University Hospital Bratislava, Bratislava, Slovakia; 45 Genetics Program, North York General Hospital, Toronto, ON Canada; 46 Institute of Human Genetics, Medical Faculty, Heinrich Heine University, Düsseldorf, Germany; 47 Institute of Neurogenomics, Helmholtz Zentrum München, Neuherberg, Germany; 48 Munich Cluster for Systems Neurology (SyNergy), Munich, Germany; 49 Neurogenetics, Technische Universität München, Munich, Germany; 50 Genetic Health Service New Zealand (Northern Hub), Auckland, New Zealand; 51 Faculty of Medical and Health Sciences, University of Auckland, Auckland, New Zealand; 52 Clinical and Translational Sciences Institute, Medical College of Wisconsin, Milwaukee, WI USA; 53 Department of Biochemistry, Medical College of Wisconsin, Milwaukee, WI USA; 54 Department of General Pediatrics, Neonatology and Pediatric Cardiology, Heinrich-Heine-University, Düsseldorf, Germany

**Keywords:** autism, developmental delay, histone acetylation, MSL3, X-linked

## Abstract

**Purpose:**

We sought to delineate the genotypic and phenotypic spectrum of female and male individuals with X-linked, *MSL3*-related disorder (Basilicata–Akhtar syndrome).

**Methods:**

Twenty-five individuals (15 males, 10 females) with causative variants in *MSL3* were ascertained through exome or genome sequencing at ten different sequencing centers.

**Results:**

We identified multiple variant types in *MSL3* (ten nonsense, six frameshift, four splice site, three missense, one in-frame-deletion, one multi-exon deletion), most proven to be de novo, and clustering in the terminal eight exons suggesting that truncating variants in the first five exons might be compensated by an alternative *MSL3* transcript. Three-dimensional modeling of missense and splice variants indicated that these have a deleterious effect. The main clinical findings comprised developmental delay and intellectual disability ranging from mild to severe. Autism spectrum disorder, muscle tone abnormalities, and macrocephaly were common as well as hearing impairment and gastrointestinal problems. Hypoplasia of the cerebellar vermis emerged as a consistent magnetic resonance image (MRI) finding. Females and males were equally affected. Using facial analysis technology, a recognizable facial gestalt was determined.

**Conclusion:**

Our aggregated data illustrate the genotypic and phenotypic spectrum of X-linked, *MSL3*-related disorder (Basilicata–Akhtar syndrome). Our cohort improves the understanding of disease related morbidity and allows us to propose detailed surveillance guidelines for affected individuals.

## INTRODUCTION

*MSL3* resides on the X-chromosome and encodes a subunit of the chromatin-associated male specific lethal (MSL) complex.^1,2^ The MSL complex mediates global histone H4 lysine-16 acetylation (H4K16ac) and plays a crucial role as an epigenetic modulator in flies and mammals.^1–4^ In mammalian species, the core MSL complex consists of MSL1, MSL2, MSL3, and MOF (males absent on the first).^2,3,5^ MSL3 itself contains a C-terminal morf-related gene (MRG) domain, responsible for MSL complex formation, and an N-terminal chromo domain (CD), implicated in chromatin targeting.^6–12^

*MSL3* was first identified as a candidate gene in the Deciphering Developmental Disorder Study^13^ and was recently described as the underlying genetic cause of Basilicata–Akhtar syndrome (MIM 301032), a novel X-linked neurodevelopmental disorder that equally affects female and male individuals.^14^ In a total of 16 patients (including two siblings), exome sequencing revealed a variety of associated variant types in *MSL3*, but mainly loss-of-function variants. Except for the siblings with suspected parental mosaicism and a maternally inherited X-chromosome inversion, all variants occurred de novo. In transfected HEK293 cells, mutant proteins displayed loss of interaction with the histone acetyltransferase MOF and with MSL1. In patient-derived cells, a bulk reduction of acetylated H4K16 compared with wild type was observed. Transcriptome analyses of patient cells showed dysregulation, mainly downregulation, of genes involved in developmental pathways. Treatment with a histone deacetylase inhibitor in vitro restored acetylation levels and *MSL3* mutant cells adopted the migratory behavior of the control cells.

Common clinical features in affected individuals include global developmental delay, delayed speech, muscular hypotonia, feeding difficulties in early infancy, progressive spasticity, and overlapping facial dysmorphism.^14^

However, with only 16 individuals described to date, the full genotypic and phenotypic spectrum of *MSL3*-related syndrome remains incompletely elucidated. Hence, this study aims to provide a comprehensive clinical and molecular characterization of individuals with causative variants in *MSL3*, to study possible genotype–phenotype correlations and to determine sex-specific differences.

## MATERIALS AND METHODS

### Participants and samples

Individuals with disease associated variants in *MSL3* were ascertained via the public data sharing platform GeneMatcher (entry “*MSL3*”)^15^ and via a Facebook family group (“MSL3 syndrome”). All individuals or their legal guardians gave written informed consent for their participation in the study and the publication of detailed clinical data. Additional informed consent was obtained when identifying images were included in this article. We obtained and thoroughly reviewed clinical records and genetic findings in every individual as well as photographs and brain magnetic resonance images (MRIs) when available. Individual 13 was previously published by Basilicata et al. (as P4)^14^ and was enrolled in this study after obtaining additional clinical follow-up data.

### Ethics statement

The study was performed in agreement with the ethical standards of the responsible committee on human experimentation (institutional and national) and with the Declaration of Helsinki, and was approved by the local ethics committee of the Technical University Munich (#5360/12S).

### Exome sequencing

Exome sequencing (ES), genome sequencing (GS), and *MSL3* Sanger sequencing was carried out independently at ten different research or diagnostic centers using genomic DNA extracted from leukocytes or whole blood. Technical details can be found in Supplementary Information. Variants’ pathogenicity was classified according to the American College of Medical Genetics (ACMG) guidelines.^16^ Nonsense, frameshift, and splice variants as well as multi-exon deletions were classified as loss-of-function variants in contrast to missense and indel variants.

### Statistics

Statistical analyses were performed in R (version 3.5.3).^17^ Comparative analyses between females and males and between loss-of-function variants and missense variants/in-frame deletions were calculated using the Fisher’s exact test.^18^ Given the exploratory design of our study, *P* values < 0.05 were considered statistically significant. Counts for clinical features are given only for those where the respective information was available, therefore the total count can be lower than the total number of individuals.

### Molecular modeling

SpliceAI (Illumin=a) was used to predict splice junctions for splice variants.^19^ For 3D modeling of identified missense variants and splice variants that are predicted to escape nonsense-mediated decay (NMD), we used the experimentally solved structure of the MSL3:MSL1 tetramer complex (PDB: 2y0n)^12^ with unresolved loops modeled using homology-based methods.^20^ Variants were mapped to the protein structure using custom scripts. ΔΔGfold was calculated for each variant studied using FoldX version 4.0.^21^

### Facial analysis

The DeepGestalt technology by Face2Gene (FDNA Inc., Boston, MA) was used to assess the presence of distinct facial patterns. Twelve frontal facial photographs (P1, P2, P3, P5, P6, P7, P8, P9, P10, P11, P12, P13) were obtained from Basilicata et al.^14^ Individuals P14 and P15 were excluded as deletions spanning more genes than *MSL3* were identified in the latter. For individual P4, who was also included in this study (as individual 13), a more recent photograph was chosen. Frontal images of additional 17 unpublished individuals from this study were available, thus adding up to a total of 30 individuals applicable for facial analysis. Age-, sex-, and ethnicity-matched controls were used as a comparison cohort. As previously reported, a composite image of the affected individuals was generated using the DeepGestalt facial analysis and binary comparison between controls and affected individuals was evaluated by measuring the receiver operating characteristic (ROC) curves and the corresponding area under the curve (AUC).^22^

## RESULTS

### Demographic features

**Fig. 1:**
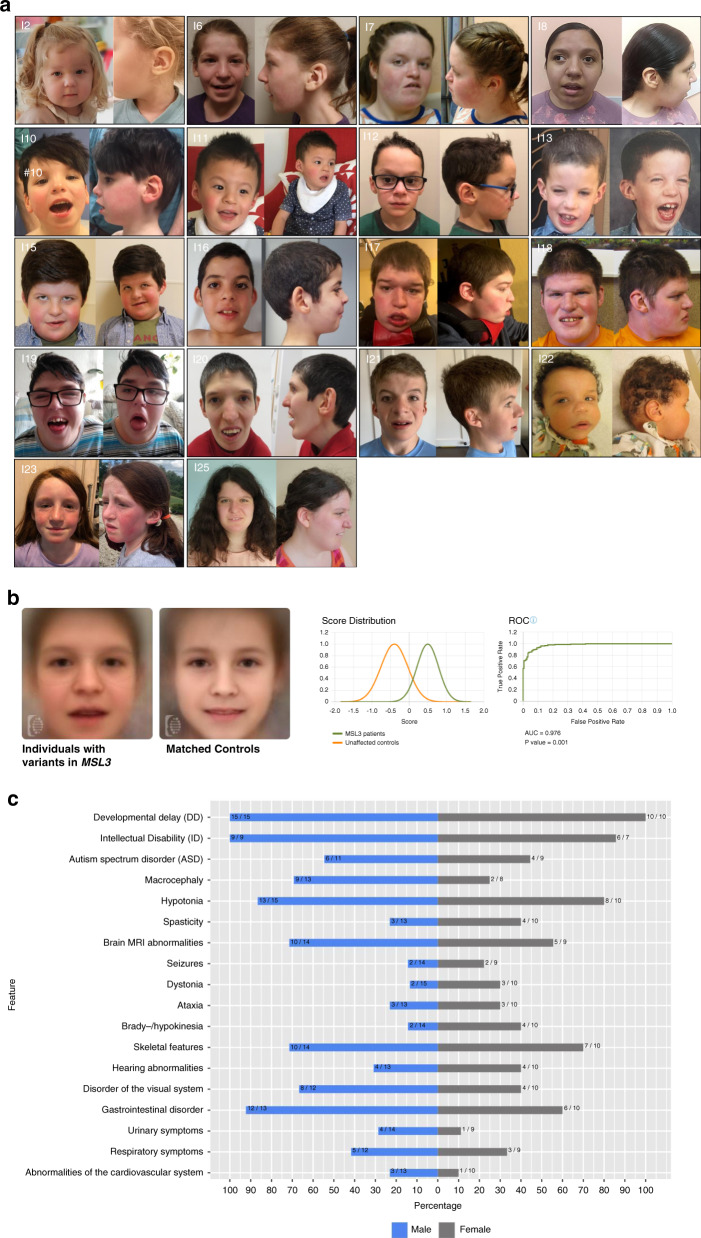
Phenotypic characteristics of males and females with X-linked, *MSL3*-associated disorder. (**a**) Facial gestalt of individuals with causative variants in *MSL3* showing dysmorphic features with broad nasal bridge, ear abnormalities (low set and/or posteriorly rotated ears, preauricular tags, overfolded helices), and coarse facial features in older individuals being the most common characteristics. (**b**) Using Face2Gene, a composite image based on the frontal facial images of previously published and newly diagnosed cases with *MSL3*-associated disorder was created. The mask of the healthy controls was generated by an age-, sex-, and ethnicity-matched control group. The composite image of individuals with *MSL3* variants visualizes dysmorphic and distinguishing facial features (round face, coarse facial features, broad nasal bridge, puffy eyes, hypertelorism, downslanting palpebral fissures, downturned corners of the mouth). The aggregated binary comparison (*AUC* area under the curve, *ROC* receiver operating characteristic) demonstrates a significant difference between the two cohorts (*p* < 0.005). (**c**) Back-to-back chart visualizes the frequency (in percent) of respective clinical features present in females (gray bars, *n* = 10) and males (blue bars, *n* = 15). The distribution of no feature was significantly different between the respective subgroups (Fisher’s exact test).

Our cohort of 25 individuals with hemizygous or heterozygous variants in *MSL3* includes 15 males and 10 females whose ages ranged from 14 months to 30 years (median: 7.25 years, interquartile range: 3.08–16.33 years) at the time of the last follow-up. Table 1 gives an overview of the clinical phenotype of the 25 individuals described in this study. Figure 1a displays the facial appearance of the individuals for whom facial pictures were available. A comprehensive summary of the clinical features of the 25 individuals is provided in Table S1.

**Table 1:** a: Clinical features of individuals 1-14. b: Clinical features of individuals 15-25 and previously published individuals with variants in *MSL3*.

Individual	1	2	3	4	5	6	7	8	9	10	11	12	13	14
Age at last assessment	14m	1y 10m	4y 1m	5y 5m	5y 7m	8y 1m	16y 4m	17y 8m	3y 1m	3y 1m	4y 2m	7y 3m	6y 3m	2y 9m
Gender	female	female	female	female	female	female	female	female	male	male	male	male	male	male
**Variant (NM_078629.4)**														
cDNA change	c.1466+1G>A	c.1146del	c.1310A>C	c.589-4_591del	c.1319dup	c.808_809del	c.(465+1_466-1)_(908+1_909-1)	c.1168_1169del	c.913C>T	c.1105C>T	c.1382-1G>A	c.1125_1141dup17	c.1370T>C	c.590_593del
Amino acid change	p.?	p.(Lys383Serfs*22)	p.(Asn437Thr)	p.?	p.(Gly441Argfs*2)	p.(Pro270Valfs*8)	p.?	p.(Lys390Glufs*6)	p.(Gln305*)	p.(Gln369*)	p.?	p.(Met381Argfs*30)	p.(Leu457Pro)	p.(Leu197*)
Zygosity	heterozygous	heterozygous	heterozygous	heterozygous	heterozygous	heterozygous	heterozygous	heterozygous	hemizygous	hemizygous	hemizygous	hemizygous	hemizygous	hemizygous
Inheritance	*de novo*	*de novo*	*de novo*	*de novo*	*de novo*	NA	*de novo*	not present in mother	*de novo*	*de novo*	maternal mosaicism	*de novo*	*de novo*	*de novo*
**Development**														
DD (+/−)	+	+	+	+	+	+	+	+	+	+	+	+	+	+
Speech delay (+/−)	+	+	+	+	+	+	+	+	+	+	+	+	+	+
Motor delay (+/−)	+	+	+	+	+	+	+	−	+	+	+	+	+	+
ID (+/−)	NA	+	+	+	NA	NA	+	+	NA	+	NA	+	NA	+
Level of intellectual disability	NA	NA	mild	NA	NA	NA	profound	moderate	NA	NA	NA	Borderline intellectual functioning	NA	NA
**Neurological findings**														
ASD (+/−)	−	−	−	−	+	NA	+	+	−	+	+	+	+	−
Seizures (+/−)	−	NA	+	−	−	−	+	−	−	−	−	−	−	−
Hypotonia (+/−)	+	+	+	+	+	−	−	+	+	+	−	+	+	+
Spasticity (+/−)	−	−	−	+	+	−	−	+	−	−	−	−	−	−
Dystonia (+/−)	−	+	−	−	+	+	−	−	−	−	−	−	−	−
Ataxia (+/−)	−	−	−	+	+	−	−	−	−	−	−	−	−	−
Brady-/hypokinesia (+/−)	+	−	−	−	−	+	−	+	−	−	−	−	−	+
**Additional findings**														
Macrocephaly (+/−)	−	−	−	NA	−	−	−	+	+	+	NA	−	+	−
Cardiovascular abnormalites (+/−)	−	−	−	−	−	+	−	−	−	−	−	−	−	−
Respiratory symptoms (+/−)	+	−	−	−	+	NA	−	−	−	+	−	−	−	+
Gastrointestinal disorder (+/−)	−	+	+	−	+	+	+	−	+	+	+	+	+	−
Disorder of the visual system (+/−)	+	−	−	−	+	−	−	+	+	+	−	+	+	−
Hearing abnormalities (+/−)	+	−	−	−	+	+	−	−	−	−	+	−	−	−
Skeletal features (+/−)	−	−	+	−	+	+	+	+	+	+	−	−	−	+
Urinary symptoms (+/−)	−	+	−	−	−	NA	−	−	−	+	−	−	−	−
Brain MRI abnormalities	+	+	+	+	−	NA	−	−	+	−	+	+	−	−

Individual	15	16	17	18	19	20	21	22	23	24	25	Our cohort (n=25)^a^	Basilicata et al^14^ (n=12)^b^	Combined (n=37)
Age at last assessment	7y 10m	9y 1m	19y 6m	19y 8m	23y	30y	9y 10m	15m	9y 9m	2y 5m	17y 2m			
Gender	male	male	male	male	male	male	male	male	female	male	female			
**Variant (NM_078629.4)**														
cDNA change	c.1362_1364del	c.1171+2_1171+4del	c.961C>T	c.1373G>T	c.1314C>A	c.590_593del	c.865A>T	c.1347C>A	c.566dup	c.973_974del	c.1372C>T			
Amino acid change	p.(Gln454del)	p.?	p.(Gln321*)	p.(Arg458Leu)	p.(Tyr438*)	p.(Leu197*)	p.(Lys289*)	p.(Tyr449*)	p.(Tyr189*)	p.(Gln326Alafs*5)	p.(Arg458*)			
Zygosity	hemizygous	hemizygous	hemizygous	hemizygous	hemizygous	hemizygous	hemizygous	hemizygous	heterozygous	hemizygous	heterozygous			
Inheritance	*de novo*	*de novo*	*de novo*	*de novo*	*de novo*	NA	*de novo*	*de novo*	*de novo*	*de novo*	*de novo*			
**Development**														
DD (+/−)	+	+	+	+	+	+	+	+	+	+	+	**25/25 (100%)**	**12/12 (100%)**	**37/37 (100%)**
Speech delay (+/−)	+	+	+	+	+	+	+	+	+	+	+	**25/25 (100%)**	**12/12 (100%)**	**37/37 (100%)**
Motor delay (+/−)	+	+	+	+	+	+	+	+	+	+	+	**24/25 (96%)**	**12/12 (100%)**	**36/37 (97%)**
ID (+/−)	NA	+	+	+	+	+	+	**NA**	−	NA	+	**15/16 (94%)**	**11/11 (100%)**	**26/27 (96%)**
Level of intellectual disability	NA	mild	severe	mild	moderate	moderate (to severe)	mild	NA	NA	NA	mild (to moderate)			
**Neurological findings**														
ASD (+/−)	−	+	+	−	NA	NA	−	NA	−	NA	+	**10/20 (50%)**	**No data available**	**10/20 (50%)**
Seizures (+/−)	NA	+	+	−	−	−	−	−	−	−	−	**4/23 (17%)**	**1/12 (8%)**	**5/35 (14%)**
Hypotonia (+/−)	+	+	+	−	+	+	+	+	+	+	+	**21/25 (84%)**	**12/12 (100%)**	**33/37 (89%)**
Spasticity (+/−)	+	−	+	+	NA	NA	−	−	+	−	−	**7/23 (30%)**	**9/12 (75%)**	**16/35 (46%)**
Dystonia (+/−)	+	−	−	−	+	−	−	−	−	−	−	**5/25 (20%)**	**No data available**	**5/25 (20%)**
Ataxia (+/−)	NA	+	+	−	+	−	−	−	−	NA	+	**6/23 (26%)**	**No data available** ^**c**^	**6/23 (26%)**
Brady-/hypokinesia (+/−)	NA	−	−	−	−	+	−	−	+	−	−	**6/24 (25%)**	**No data available**	**6/24 (25%)**
**Additional findings**														
Macrocephaly (+/−)	+	+	+	+	NA	−	+	−	+	+	NA	**11/21 (52%)**	**5/12 (42%)**	**16/33 (48%)**
Cardiovascular abnormalites (+/−)	+	−	−	−	NA	−	+	NA	−	+	−	**4/23 (17%)**	**3/11 (27%)**	**7/34 (21%)**
Respiratory symptoms (+/−)	NA	+	+	−	NA	−	NA	−	−	+	+	**8/21 (38%)**	**3/9 (33%)**	**11/30 (37%)**
Gastrointestinal disorder (+/−)	NA	+	+	+	NA	+	+	+	+	+	−	**18/23 (78%)**	**10/12 (83%)**	**28/35 (80%)**
Disorder of the visual system (+/−)	NA	−	+	+	NA	+	−	+	+	NA	−	**12/22 (55%)**	**No data available** ^**d**^	**12/22 (55%)**
Hearing abnormalities (+/−)	+	−	−	+	NA	−	+	−	+	NA	−	**8/23 (35%)**	**1/11 (9%)**	**9/34 (26%)**
Skeletal features (+/−)	+	+	+	+	NA	+	+	−	+	+	+	**17/24 (71%)**	**9/12 (75%)**	**26/36 (72%)**
Urinary symptoms (+/−)	+	+	+	−	NA	−	−	−	−	−	−	**5/23 (22%)**	**2/12 (17%)**	**7/35 (20%)**
Brain MRI abnormalities	+	+	+	+	NA	+	+	+	+	−	−	**15/23 (65%)**	**5/12 (42%)**	**20/35 (57%)**

Abbreviations: DD = developmental delay; ID = intellectual disability; NA = not available; ASD = autism spectrum disorder; MRI = magnetic resonance imaging

^a^Counts for clinical features are given only for those where the respective information was available, therefore the total count can be lower than the total number of individuals

^b^Two individuals with deletions spanning more genes than *MSL3*, one individual with an inherited inversion as well as one individual (reported as P4) who was included in this study as individual 13 were excluded.

^c^Three individuals were reported to have an ataxic and broad-based gait.

^d^One individual was reported to have retinal abnormalities.

### Neurological findings

Neurological abnormalities constituted the main clinical phenotype of *MSL3*-related disorder. Developmental delay (DD) was prominent in all individuals (*n* = 25/25, 100%), with global DD in 24/25 (96%) individuals and isolated speech development delay in 1/25 (4%) individual. Fifteen of 16 (94%) individuals were diagnosed with intellectual disability (ID). ID was classified as mild in 5/15 (33%) individuals, moderate in 3/15 (20%) individuals, severe in 1/15 (7%) individuals, profound in 1/15 (7%) individuals, and borderline intellectual functioning in 1/15 (7%) individuals. Four of 15 (27%) individuals had received the diagnosis of ID; however, the level of ID had not been determined.

Twenty-two of 25 (88%) individuals had a history of abnormal muscle tone, comprising truncal muscular hypotonia (*n* = 21) and/or spasticity of the upper and lower limbs (*n* = 7) while 5 individuals had mixed tone. Muscular hypotonia was predominantly evident in infancy, while spasticity appeared to evolve with increasing age.

A heterogeneous spectrum of movement disorders was reported including dystonia (*n* = 5/25; 20%), brady-/hypokinesia (*n* = 6/24; 25%), and ataxia (*n* = 6/23; 26%). Dystonic movements were described as generalized as well as focal. Three individuals had received the clinical diagnosis of cerebral palsy before genetic testing was initialized.

Most individuals ambulated prior to age of 3 years with the exception of one individual who did not walk until the age of 4 years. Three individuals (ages: 14 months, 15 months, 33 months) were still non-ambulatory at the time of last follow-up. Gait abnormalities were present in eight individuals. One individual (individual 17) developed progressive gait disturbance due to ataxia by the age of 13 years and was non-ambulatory by the age of 15 years. No other individual was reported to have lost ambulation.

Receptive language skills seemed to be better developed than expressive language; however, these observations were not based on standardized testing. Ten of 25 (40%) individuals were nonverbal at the time of data collection (ages: 14 months, 15 months, 29 months, 33 months, 3 years, 3 years, 4 years, 8 years, 16 years, 19 years).

None of the participants showed signs of developmental regression except for two individuals. Individual 18 displayed two episodes of developmental regression in his verbal and cognitive abilities at ages 13 and 18 years from which he recovered only partially. Neither of these episodes was associated with a documented triggering event. Individual 17 had regression of his motor skills with progressive gait disturbance and became nonambulatory. Ten of 20 (50%) individuals had a diagnosis of autism spectrum disorder (ASD). Other behavioral abnormalities included attention deficit–hyperactivity disorder (ADHD) (*n* = 4), aggressive behavior (*n* = 2), anxiety (*n* = 4), obsessive–compulsive disorder (*n* = 2), and self-injurious behavior (*n* = 2). Three individuals, however, were described as very friendly and sociable.

Four of 23 (17%) individuals had a history of seizure(s). Seizures were well controlled with antiepileptic treatment in all cases. Individual 17, who experienced a single seizure in infancy, later stopped antiepileptic drugs and has been seizure-free since then.

### MRI findings

**Fig. 2:**
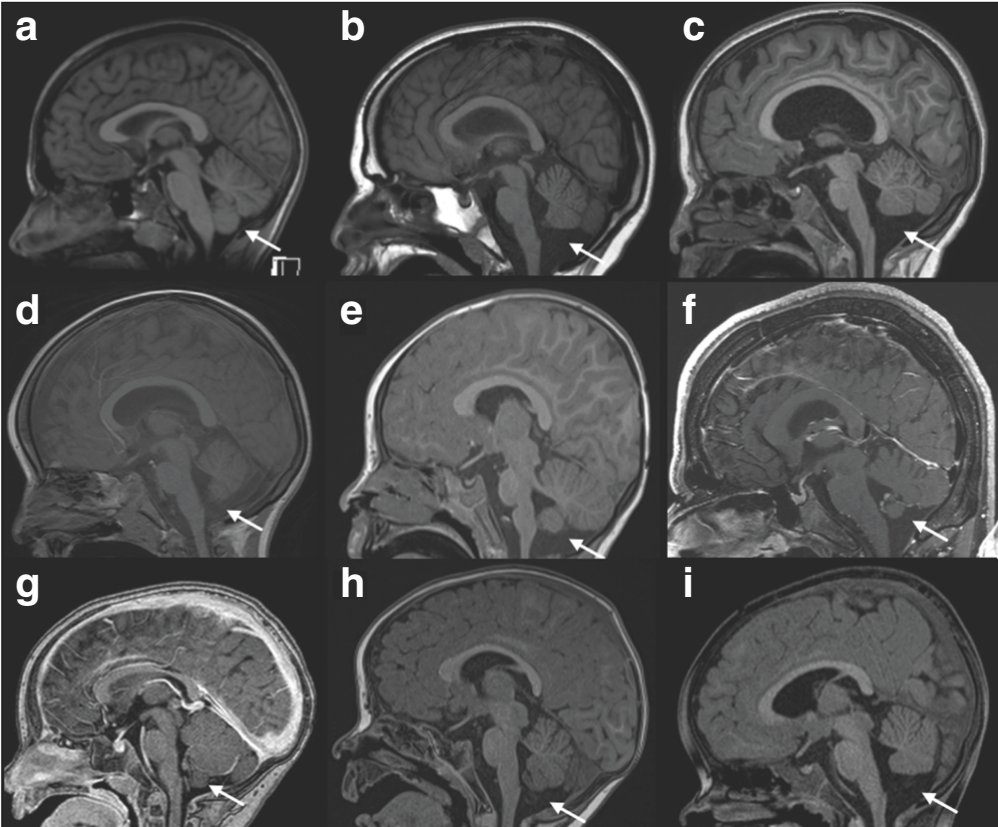
Brain magnetic resonance image (MRI) findings in individuals with pathogenic variants in *MSL3*. (**a**) Brain MRI image (T1-weighted sagittal view) of a healthy individual at the age of 13 years. The white arrow indicates the inferior part of the cerebellar vermis. (**b**–**i**) Brain MRI images (sagittal views) of individuals with variants in *MSL3* (T1-weighted sequences; [**f**, **g**] are contrast enhanced sequences). (**b**) Individual 17 (age 13 years). (**c**) Individual 15 (age 7 years). (**d**) Individual 16 (age 5 years). (**e**) Individual 11 (age 24 months). (**f**) Individual 18 (age 18 years). (**g**) Individual 3 (age 14 months). (**h**) Individual 1 (age 12 months). (**i**) Individual 2 (age 20 months). In all individuals with pathogenic variants in *MSL3* variable hypoplasia (or atrophy) of the inferior part of the cerebellar vermis was observed (white arrows; please compare with normal findings in [**a**]).

To gain further insight into brain MRI abnormalities, relevant images or whole MRI data sets of eight affected individuals were evaluated by the same pediatric neuroradiologist (individuals 1, 2, 3, 11, 15, 16, 17, and 18). Image analysis revealed hypoplasia of the inferior part of the cerebellar vermis as a consistent feature in the individuals investigated (Fig. 2). Variable dilatation at the trigone of the lateral ventricles was also noted (Fig. S1). In addition, a spectrum of variable central nervous system (CNS) findings was reported including nonspecific white matter abnormalities (five individuals) and moderate widening of external cerebrospinal fluid spaces (two individuals). Follow-up MRIs were available for two individuals (2 and 17). In these cases, cerebellar abnormalities were nonprogressive (Fig. S2).

### Additional findings

Macrocephaly (occipital frontal circumference [OFC] > 97th percentile) (including relative macrocephaly) was present in 3/14 (21%) individuals at birth, while at the time of the last follow-up macrocephaly was seen in 11/21 (52%) individuals indicating secondary macrocephaly. Birth length was within normal limits (3rd–97th percentile) in 19/19 (100%) individuals and 4/23 (17%) individuals subsequently developed short stature (<3rd percentile). Polyhydramnios was evident in 4/18 (22%) pregnancies. A heterogeneous spectrum of gastrointestinal problems, mainly constipation and gastroesophageal reflux disease, was found in 18/23 (78%) individuals. Five of 23 (22%) individuals were reported to have urinary symptoms: two individuals had urinary retention needing catheterization, one individual had bilateral hydroceles at birth, and two had hydronephrosis and pyeloureteral junction stenosis, respectively. Respiratory symptoms were observed in 8/21 (38%) individuals and 4/23 (17%) individuals had abnormalities of the cardiovascular system. Vision abnormalities were common but heterogeneous (12/22; 55%). Eight of 23 (35%) individuals had conductive and/or sensorineural hearing loss (uni- or bilateral). Individual 6, who had moderate to severe bilateral sensorineural deafness, also harbored a pathogenic homozygous *STRC* gene deletion as an additional genetic finding and was diagnosed with deafness, autosomal recessive 16 (MIM 603720). We considered it more likely that the pathogenic variant in *STRC* primarily contributed to the hearing impairment in this individual. Skeletal features (e.g., pes planus, plagiocephaly, pectus carinatum) were evident in 17/24 (71%) individuals. Abnormalities of the fingers, such as clinodactyly, tapered and slender fingers, which were observed in several individuals, are depicted in Fig. S3. A detailed description of additional findings is provided in the Supplementary case reports and Table S1.

### Facial appearance

Dysmorphic facial features were reported in the majority (*n*=23/24 [96%]). Figure 1a displays the facial appearance of 18 individuals for whom facial pictures were available. Dysmorphic features were relatively mild, especially at a young age, but commonly shared features were round face, broad nasal bridge, epicanthal folds, hypertelorism, prominent forehead, and coarse facial features. Additional features included arched brows, high nasal bridges, abnormal dentition, overbites, narrow palates, and micrognathia. An abnormal shape or position of the outer ear encompassing low set and/or posteriorly rotated ears, preauricular tags, and overfolded helices was present in 14/22 (64%) individuals.

We used Face2Gene RESEARCH application to perform computer-based facial analyses of 30 individuals with *MSL3* variants (12 individuals from Basilicata et al.,^14^ 18 individuals from this study). The binary comparison between individuals with *MSL3* variants and unaffected controls (age, sex, and ethnicity matched) yielded an average AUC of 0.976 expressing a significant difference between these two cohorts (*p* < 0.005) (Fig. 1b). The aggregated composite facial image/mask depicts a distinct facial gestalt (round face, coarse facial features, broad nasal bridge, puffy eyes, hypertelorism, downslanting palpebral fissures, downturned corners of the mouth) (Fig. 1b).

### Phenotypic differences between females and males

We further evaluated if specific clinical characteristics significantly (Fisher’s exact test) appear more often in females versus males (Table S4). No significant difference regarding the frequency of clinical features was found between the two groups. Figure 1c visualizes the comparison of the clinical features between females and males.

### Phenotypic differences between individuals with missense variants/in-frame deletions and individuals with loss-of-function variants

We further evaluated if specific clinical characteristics significantly (Fisher’s exact test) appeared more often in individuals with loss-of-function variants versus individuals with missense variants/in-frame deletions (Table S4). No significant difference regarding the frequency of clinical features was found between the two groups. Figure S4 visualizes the comparison of the clinical features between both groups.

### Variants in affected individuals

We identified heterozygous and hemizygous variants in *MSL3* (NM_078629.4) in 25 individuals from 25 independent families and observed a spectrum of variant types with loss-of-function variants being the predominant variant type (*n* = 21/25). These comprised ten nonsense variants, six frameshift variants, four splice site variants, and one multi-exon deletion. In two canonical splice site variants, exon skipping or the use of a cryptic splice site would disrupt the reading frame. The variant c.1382–1G>A is predicted to result in an in-frame product with use of a cryptic splice site (p.[Val461_Pro464del]).^19^ The splice variant c.1466+1G>A is predicted to lead to exon skipping with the introduction of a premature translation termination codon in the last exon (p.[Val461Glyfs*3]) and would most likely not undergo NMD.^23,24^ The multi-exon deletion comprises exons 6–8 and is predicted to result in the disruption of the reading frame (out-of-frame deletion) as well. All other truncating variants occurred before the last exon of *MSL3* and were predicted to undergo NMD.^23,24^ Furthermore, three missense variants as well as one in-frame deletion were detected that all affected highly conserved amino acids in the MRG domain. All detected variants were absent from gnomAD.^25^ All variants were classified as likely pathogenic or pathogenic, respectively, accordingly to the ACMG criteria (Table S2).^16^

**Fig. 3:**
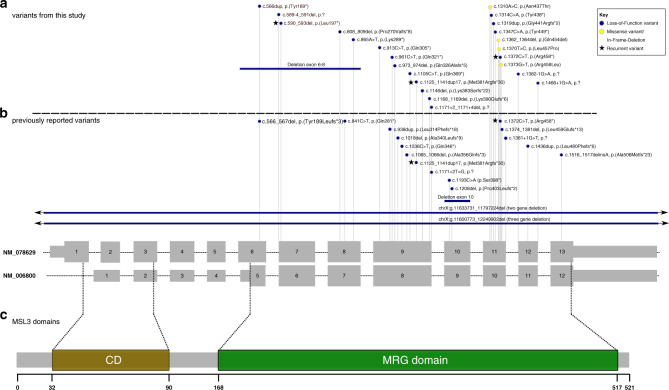
Variant type and exon distribution of variants in *MSL3* (NM_078629.4). (**a**, **b**) Gray boxes illustrate the coding sequence, smaller gray boxes illustrate untranslated regions (UTRs). The predominant *MSL3* transcript NM_078629 (ENST00000312196) and the shorter transcript NM_006800 (ENST00000380693) are shown with numbered exons. Variants identified in this study are depicted above the dotted line (**a**) and previously reported variants are presented below (**b**). The color of the circle represents the corresponding variant type (see key). Overall, the majority of variants are predicted loss-of-function variants. Three recurrent variants (depicted with stars) were observed. Notably, all newly and previously reported variants (excluding deletions spanning more genes than *MSL3*) are confined to the last eight exons (exons 6–13) of *MSL3* (NM_078629). No variants affecting only the first five exons of *MSL3* (NM_078629) were identified. (**c**) Schematic representation of MSL3 protein domains. The chromo domain (CD) (amino acid position 32–90) is depicted in brown and the MRG domain (amino acid position 168–517) is depicted in green. The shorter transcript NM_006800 corresponds only to the MRG domain.

To date, three recurrent variants have been observed: the variant c.590_593del, p.(Leu197*) was found in two unrelated individuals from this study (individuals 14 and 20), and the variants c.1125_1141dup17, p.(Met381Argfs*30) and c.1372C>T, p.(Arg458*), which were identified in individuals 12 and 25, respectively, were previously reported.^14^ All variants were located within the last eight exons (exon 6–13) of *MSL3* (NM_078629.4), encoding the MRG domain, whereas no variants were detected within the first six exons. Figure 3a visualizes the type and distribution of the variants described in this study. We furthermore aggregated all so-far reported causative variants^14,26^ in *MSL3* confirming that pathogenic variants in *MSL3* cluster in the exons encoding the MRG domain. Figure 3b illustrates the distribution of the respective variants. Table S3 summarizes all previously published variants as well as variants from this study (*n* = 41).

Parental DNA samples were available for 22 individuals. Variants were proven to be de novo in 21 of those individuals. In the healthy mother of individual 11, segregation study (Sanger sequencing) suggested the variant as low-level mosaic in blood cells. The variant load of approximately 8% was estimated using droplet digital polymerase chain reaction (ddPCR). No respective parental DNA samples were available for individuals 6, 8, and 20; however, unremarkable family histories in combination with the finding of pathogenic variants in those cases suggested likely de novo origins as well. Overall, we did not identify familial cases.

### Molecular modeling reveals common underlying mechanism

**Fig. 4:**
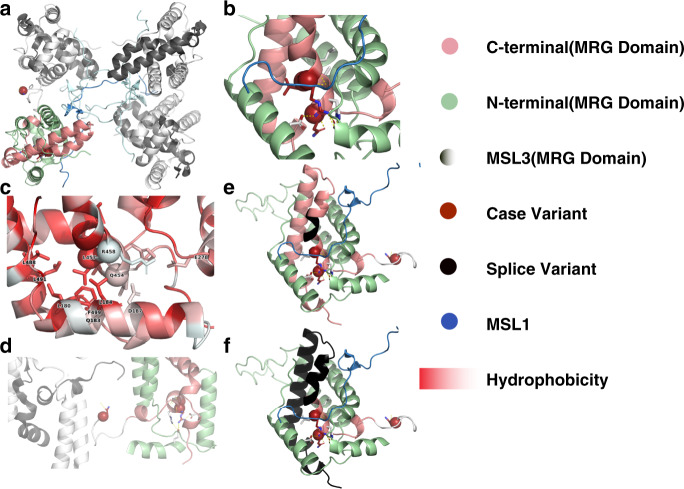
Overview of the position of case variants on the MSL3–MSL1 tetrameric complex. (**a**) MSL3–MSL1 tetrameric complex with the N-terminal and C-terminal MRG regions from one monomer highlighted in pale green and salmon. The sites of missense variants are marked by red spheres. p.(Asn437Thr) is the first amino acid in the disordered loop and is present at the interface of the tetramer. p.(Leu457Pro) and p.(Arg458Leu) are located within the bipartite fold region. (**b**) The environment around the case variants is shown in sticks. The Arg458 residue interacts with Asp187 and Glu278 of the N-terminal region of the MRG domain. (**c**) The structure is highlighted based on hydrophobicity which ranges from red (hydrophobic) to white (hydrophilic). It is visually evident that one side of this region is highly hydrophobic and includes Leu457, while the other side is hydrophilic and includes Arg458 and Gln454. (**d**) Variant Asn437Thr is marked by a red sphere, present at the monomer–monomer interface. (**e**, **f**) The amino acids removed by splice variants are colored in black and affect the same region as missense variants.

We investigated the 3D pattern of genetic variants on the encoded protein of the MRG domain. The MRG domain is predicted to make a bipartite fold where the N- and C-terminal regions intertwine. The substitutions p.(Leu457Pro) and p.(Arg458Leu), the in-frame deletion p.(Gln454del), as well as the two splice variants with the predicted consequences p.(Val461_Pro464del) and p.(Val461Glyfs*3) that presumably escape NMD all occur in the middle of this region (Fig. 4). These alterations will, most likely, destabilize the fold, which is necessary for binding to MSL1. The folding energy (ΔΔG_fold_) is highly destabilizing for missense variants p.(Leu457Pro) (5.36 kcal/mol) and p.Arg458Leu (4.02 kcal/mol), while it is neutral for the variant p.(Asn437Thr) (−0.23 kcal/mol) in the monomer. The p.(Asn437Thr) variant is present at the interface of the complex, possibly interfering with the complex formation.

## DISCUSSION

Here, we present 25 individuals (15 male, 10 female) with disease-causing hemizygous or heterozygous variants in *MSL3*, which is the largest cohort of individuals with X-linked, *MSL3*-related disorder to date. With the exception of one individual whose phenotypically normal mother had germline mosaicism, all cases were either proven (*n* = 21) or presumed to be de novo based on family history (*n* = 3).

Our cohort allowed a more comprehensive definition of X-linked, *MSL3*-related disorder as a syndromic condition affecting females and males equally, with varying degrees of ID (mild to severe) and DD as the most consistent features. ASD, found in 50% of all individuals, emerged as a new feature of X-linked, *MSL3*-related disorder. Variable neurological findings were common and comprised muscular hypotonia, upper and lower limb spasticity, abnormal movements (dystonia, ataxia, hypokinesia), and cerebellar vermis hypoplasia as a consistent MRI finding. Data for longitudinal MRI studies were only available for two individuals (Fig. S2) and we can therefore not completely exclude the possibility that the cerebellar vermis volume loss is progressive (atrophy). Interestingly, the cerebellar abnormalities were only identified during reanalysis for several cases and comparison with other individuals with *MLS3* variants, highlighting the importance of systematic image analysis in rare neurological disorders.

A high prevalence of features affecting other organ systems was encountered, some of which occurred more frequently than previously reported in the existing literature. Gastrointestinal problems (constipation, gastroesophageal reflux, and vomiting) as well as disorders of the genitourinary system were commonly found. Hearing impairment, namely sensorineural and/or conductive hearing loss, macrocephaly, as well as a variety of visual abnormalities were observed as new significant features. Polyhydramnios also emerged as a newly recognized prenatal feature in *MSL3*-related disorder.

Facial dysmorphism, which appeared rather subtle in the younger individuals, was described in the majority of individuals and included several consistent facial features. Computational facial analysis technology was able to accurately discriminate between affected individuals and healthy controls, indicating that affected individuals have a recognizable facial gestalt that might help to diagnose affected individuals early using “next-generation phenotyping” or to guide diagnosis in case of inconclusive genetic results.^27^

Among 25 disease-causing variants identified in this cohort, 22 variants were novel. Using ACMG criteria, all variants were classified as likely pathogenic or pathogenic (Table S2). Besides considerably expanding the list of likely pathogenic and pathogenic variants in *MSL3*, we illustrate a variety of variant types to be associated with X-linked, *MSL3*-related disorder, with loss-of-function variants accounting for the majority (*n* = 21/25). Loss-of-function variants and missense variants were found equally in affected males and females. Prior to this study, only one individual harboring a disease-causing missense variant—previously referred to as p.(Leu308Pro) and listed here as p.(Leu457Pro)—in *MSL3* had been reported.^14^ Of note, this individual was also included in this study (as individual 13) after obtaining additional follow-up data. Herein, we describe two additional missense variants (p.[Asn437Thr] and p.[Arg458Leu]) as well as one in-frame deletion (p.[Gln454del]) confirming that missense variants and in-frame deletions are part of the genotypic spectrum of *MSL3*-related disorder. Whereas the missense variant p.(Asn437Thr) is predicted to impair complex formation with MSL1 the substitutions p.(Leu457Pro), and p.(Arg458Leu) as well as the in-frame deletion p.(Gln454del) all affect highly conserved amino acids disrupting the bipartite fold region of the MRG domain.^12,28^ The lack of clinical differences between individuals with loss-of-function and missense variants in our cohort (Fig. S5, Table S4) points toward a loss of function as the mutational mechanism. However, this analysis has a low power and larger studies might identify subtle differences between these groups.

All currently reported causative variants (excluding multigene deletions) (*n* = 41) were confined to the last eight exons (exon 6–13) of *MSL3* with exon 9 (*n* = 14) and exon 11 (*n* = 11) being most frequently affected whereas no pathogenic variants were observed in the first exons in individuals with *MSL3*-associated disease (Table S3).^14,26^ The latter finding might indicate that translation of the shorter transcript NM_006800 (ENST00000380693), which lacks the CD and is only comprised of the MRG domain responsible for interaction with MSL1, can rescue gene function (functional MSL complex) even in the presence of variants in the first exons. These findings suggest that the N-terminal CD of MSL3 is either redundant or that a mutant CD is associated with traits different than those seen in Basilicata–Akhtar syndrome. One case (nsv1075719) with a deletion comprising the first exon is listed in the Database of Genomic Variants (DGV) and an additional case with a heterozygous frameshift variant p.Lys34ArgfsTer20 in the first exon in the longer transcript NM_078629 is listed in gnomAD, compatible with the hypothesis that the CD is redundant.^25,29^ However, it should be noted that the variant listed in gnomAD has low coverage and poor base quality. Another explanation for the observed pattern is that loss-of-function variants in the first five exons are not viable and that loss-of-function variants in the eight C-terminal exons escape NMD and result in the translation of a truncated protein with residual function. However, the full gene deletion argues against this possibility.^25,29^

It should be noted that our cohort contains one individual (individual 11) with parental germline mosaicism (variant allele fraction of 8% in a phenotypically normal mother). This represents the first genetically confirmed case of parental germline mosaicism in the context of X-linked, *MSL3*-related disorder.

*MSL3* was recently found to escape X-chromosome inactivation (XCI).^30^ X-linked genes that largely escape from XCI (escapees) have been linked to genetic disorders that affect both female and male individuals, however, with sex-specific differences.^30–34^ We therefore conducted a comparative analysis between females and males to evaluate possible sex-specific differences (Table S4). Our cohort included 15 males and 10 females with disease-causing variants in *MSL3* and we did not identify significant differences regarding the frequency of clinical features between the two groups. The absence of obvious differences between males and females is so far unexplained and future studies are required to elucidate the precise molecular basis behind this phenomenon.

Even though specific treatment is not available to date, we observed a large number of disease-associated complications, which can be managed symptomatically. Based on the clinical phenotypic delineation in this *MSL3*-associated disorder cohort, we propose the following clinical management and follow-up recommendations. Following diagnosis, affected individuals should be referred to the following: (1) a (pediatric) ophthalmologist as impairment of the visual system was frequent and early therapy may be required to avoid amblyopia; (2) a (pediatric) otolaryngologist as hearing impairment should be treated early with hearing aids or cochlear implants; (3) gastroenterological evaluation for feeding problems and gastroesophageal reflux disease; (4) nephrology assessment including kidney ultrasound is recommended to detect structural abnormalities early that may predispose to urinary tract infections and/or subsequent renal damage; prophylactic treatment and follow-up by a renal specialist may be indicated; (5) evaluation of the skeletal system for malformations, which may require orthopedic management; and (6) regular neurodevelopmental and neurological follow-up because developmental delay, behavioral abnormalities, movement disorders, and seizures contributed most to disease burden.

## Supplementary information

Supplementary Information

Supplementary Table S1
