# Supplementary material for: Defining the genotypic and phenotypic spectrum of X-linked MSL3-related disorder
Source: Genet Med. 2020 Nov 11;23(2):384–95. doi: 10.1038/s41436-020-00993-y (PMC7862064; doi:10.1038/s41436-020-00993-y)
Supplement: Supplementary file 1 — Supplementary Information [file 41436_2020_993_MOESM1_ESM.pdf]

Defining the genotypic and phenotypic spectrum of X-linked, *MSL3*-related disorder

Supplementary Information

Supplementary Methods

Targeted sequencing methods.

Individuals 1, 19, 20 – Trio exome sequencing (individuals 1, 19) or single exome sequencing (individual 20) was performed using a Sure Select Human All Exon 60Mb V6 Kit (Agilent) for enrichment and a *Illumina NovaSeq6000* system (Illumina, San Diego, California, USA). Reads were aligned to the UCSC human reference assembly (hg19) with BWA v.0.5.8.[1] More than 97%/98% of the exome was covered at least 20× and the average coverage was more than 115×. Single-nucleotide variants (SNVs) and small insertions and deletions were detected with SAMtools v.0.1.7. Copy number variations (CNVs) were detected with ExomeDepth and Pindel.[2,3] Variant prioritization was performed based on an autosomal recessive (MAF <0.1%) and autosomal dominant (de novo variants, MAF <0.01%) inheritance.

Individual 2 - Genomic DNA was extracted from peripheral blood samples using QiAexpert. Trio WES with DNA samples of patient and healthy parents was prepared for sequencing using SureSelectXT Automation Reagent Kit and SureSelectXT Human All Exon v5 RNA probes (50Mb) (Agilent Technologies). During this process, the gDNA is sheared into smaller fragments (~300bp), adapters are added at each end and the exonic and flanking splice junctions regions of the genome are captured. PCR is also performed before and after hybridization. Sequencing was done on a HiSeq4000 Illumina system with 150bp paired-end reads. Reads were aligned to human genome build GRCh37/UCSC hg19, and analyzed for sequence variants using an in-house analysis tool, ELLA (http://allel.es/).

Individual 3 - Genomic DNA was extracted from peripheral blood samples using standard procedure. Trio WES with DNA samples of patient and healthy parents was performed as described before.[4] Briefly, the exonic and flanking splice junctions regions of the genome were captured using the Clinical Research Exome v.2 kit (Agilent Technologies, Santa Clara, CA). Sequencing was done on a NextSeq500 Illumina system with 150bp paired-end reads. Reads were aligned to human genome build GRCh37/UCSC hg19, and analyzed for sequence variants using a custom-developed analysis tool

(1). Additional sequencing technology and variant interpretation protocol have been previously described (1). Coverage on target for the index was ≥ 10x for 98.2% with a mean coverage of 198X

Individuals 4, 5, 6, 8, 9, 12, 13, 15, 17, 18, 21, 22, 23 - Using genomic DNA from the proband or proband and parent(s), the exonic regions and flanking splice junctions of the genome were captured using the SureSelect Human All Exon V4 (50 Mb), the Clinical Research Exome kit (Agilent Technologies, Santa Clara, CA) or the IDT xGen Exome Research Panel v1.0. Massively parallel (NextGen) sequencing was done on an Illumina system with 100bp or greater paired-end reads. Reads were aligned to human genome build GRCh37/UCSC hg19 and analyzed for sequence variants using a custom-developed analysis tool. Additional sequencing technology and variant interpretation protocol has been previously described.[5] The general assertion criteria for variant classification are publicly available on the GeneDx ClinVar submission page (http://www.ncbi.nlm.nih.gov/clinvar/submitters/26957/).

Individual 7, 24 - Using genomic DNA, the coding regions of targeted genes plus ~10 bases of non-coding DNA flanking each exon were captured using Agilent Clinical Research Exome hybridization probes. Captured DNA was sequenced using Illumina's Reversible Dye Terminator (RDT) platform NovaSeq 6000 using 150 by 150 bp paired end reads (Illumina, San Diego, CA, USA). The following quality control metrics are generally achieved: >98% of target bases are covered at >20x, and mean coverage of target bases >120x. Data analysis and interpretation is performed by the internally developed software Titanium-Exome. In brief, the output data from the NovaSeq 6000 is converted to fastqs by Illumina Bcl2Fastq v2. 19.0.316, and aligned by BWA mem. Variant calls are made by the GATK Haplotype caller and annotated using in house software and SnpEff. Variants are filtered and annotated using VarSeq (www.goldenhelix.com). Common benign and low quality variants are filtered from analysis.

Individual 10 - DNA samples were analyzed by Whole-Exome-Sequencing at the Institute for Genomic Medicine, Columbia University Medical Center, using the Illumina NovaSeq 6000 platform and KAPA Biosystem's library preparation kits with whole-exome capture performed using NimbleGen SeqCap EZ version 3.0. Bioinformatics processing included read mapping (Illumina DRAGEN Bio-IT Platform v.2.5.1 to GRCh37 hg19), variant calling (GATK best practices protocol), variant calling (GATK-3.6), and variant annotation (ClinEff-1.0 with Ensembl-GRCh37.73 and dbSNP annotations). Candidate pathogenic variants were evaluated using a trio analysis framework which identifies qualifying genotypes after accounting for parental genotypes and genotypes present within an in-house variant database and external control reference samples.

Individual 11 - Total genomic DNA was extracted from the biological sample using a spin column method. DNA quality and quantity were assessed through gel electrophoresis and fluorometric analysis, respectively. DNA sample was then shipped to BGI* where whole exome capture and sequencing were performed. Exome enrichment was done, using Agilent SureSelect Human All Exon V6 kits. Libraries were sequenced on Illumina HiSeq platforms to a coverage >100x. Clean sequence reads of each sample was mapped to the human reference genome (GRCh37/hg19). Burrows-Wheeler Aligner (BWA) software was used to do the alignment. Local realignment around indels and base quality score recalibration were performed using GATK, with duplicate reads removed by Picard tools. Data analysis was performed at Blueprint Genetics. The exome variant data of the patient was filtered based on all possible modes of inheritance of the disorder.

Individual 14 - Double stranded capture baits against approximately 36.5MB of the human exome (targeting >98% of the coding RefSeq and Gencode v28 regions, which was obtained from the human genome build CRCH37/hg19 on May 2018) were used to enrich target regions from fragmented genomic DNA with the Twist Human Core Exome Plus kit. The generated library was sequenced on an Illumina platform to obtain at least 20x coverage depth for >98% of the targeted bases. An in-house bioinformatics pipeline, including read alignment to GRCH39/hg19 genome assembly, variant calling and annotation, and comprehensive variant filtering was applied. All disease causing variants reported in HGMD®, in ClinVar and in CentoMD® as well as all variants with a minor allele frequency (MAF) below 1% in the gnomAD database are considered. The investigation for relevant variants is focused on coding exons and flanking +/- 20 intronic bases. All potential modes of inheritance are considered. In addition, provided family history and clinical information are used to evaluate identified variants with respect to their pathogenicity and causality, and are categorized into classes 1-5. All variants related to the phenotype of the patient, except benign or likely benign variants, are reported.

Individual 16 - Trio-exome sequencing: SureSelect Human All Exon V6 (Agilent) target enrichment kits, and sequencing was performed on a HiSeq4000 platform (Illumina), using paired-end reads. After removal of duplicate reads, sequences were aligned to GRCh38, and variant quality assessment, filtering and prioritization was done using an in-house developed processing pipeline based on inheritance, variant frequency in control databases, and predicted functional impact. Variants of interest were confirmed by Sanger sequencing.

Individual 25 – Whole genome sequencing (WGS) was performed using the Illumina TruSeq DNA PCR-Free sample preparation kit (Illumina, Inc.) and an Illumina HiSeq 2500 sequencer, generating a mean depth of 30×. WGS reads were aligned using Isaac Genome Alignment Software and single variant nucleotides and indels called for chromosomes 1 to 22, X, and the mitochondrial genome using the Platypus variant caller, details as outlined in previous publication.[6]

Genomics England bioinformatics tiers single nucleotide variants (SNVs) in the applied panels into Tier 1 high impact variants such as truncating variants and Tier 2 moderate impact variants such as missense changes. The remaining rare variants are in Tier 3 or untiered. The Exomiser tool is also applied to the data irrespective of panel. This tool uses HPO terms to prioritize variants according to frequency, pathogenicity, inheritance pattern of the variants and similarity of the patient phenotypes to reference genotype to phenotype associations in known human diseases, model organisms and protein-protein interactions to known human disease genes. Each variant is scored and ranked with the highest rank being 1.

Determination of mosaicism of variants

Droplet digital PCR (ddPCR) specific to NM_078629.3 (*MSL3*) was used to assess allele specific copy number at *MSL3*: c.1382-1G>A. ddPCR was performed in the presence of a fluorescent interchelating dye, EvaGreen (BioRad), using the manufacturer's recommended conditions. Quantitation was carried out by normalizing the mutant allele to the wildtype allele. In a known hemizygous control (our ref 19FH3585K) this ratio was 100%. Samples were repeated in quadruplicate.

Supplementary Case Reports

Individual 1 - This girl is the 3rd child of healthy non-consanguineous parents with German-Turkish ancestry. Two older siblings are healthy. Pregnancy was complicated by polyhydramnios. Moreover, during prenatal ultrasound a shortening of fetal extremities was suspected. The child was born at 39+4 gestational weeks (weight 3232 g, lengths 51 cm, Head circumference 36 cm). Postnatally she had respiratory problems and neonatal pneumonia was suspected. The child was transferred to the ICU and received high-flow breathing support. During the following days the condition rapidly improved and the child was discharged home. At the age of 3 months the parents noted that the child was very unsettled with frequent crying episodes. Moreover, fixation was inconstant and she interacted less with the parents compared to the healthy siblings. During the following months developmental delay became evident. Diagnostic work-up with brain MRI revealed mild widening of inner cerebrospinal fluid spaces. Muscle ultrasound showed atrophy and increased fat content of her thighs. Because of lacking speech development, she was seen by the otorhinolaryngologists who diagnosed a hearing impairment (conductive hearing loss). Eye examination indicated cortical visual impairment. Laboratory work-up, including screening for different metabolic diseases, showed no abnormalities. At the current age of 14 months the girl shows clear motoric developmental delay with truncal muscular, hypotonia and reduced spontaneous movement pattern. She is unable to crawl or to sit without support and she shows no interest in grasping objects. So far there is no speech development and she produces hardly any babbling sounds. Her current growth parameters are as follows: weight 9.9 kg (51st P), lengths 75.2 cm (18th P), head circumference 46.7 cm (63rd P). Trio exome sequencing revealed a *de novo* heterozygous splice variant c.1466+1G>A, p.?) in *MSL3* (NM_078629.3). The variant was classified as pathogenic according to the ACMG criteria.

Individual 2 – Individual 2, a girl and the second child of unrelated, healthy Norwegian parents, was born GW after a normal pregnancy at 38+6 weeks of gestation with birth measurements within the normal range. The postnatal period was unremarkable. At the age of 10 months, she presented with gastroenteritis-like symptoms (vomiting, but no diarrhoea). She was hypotonic and was admitted to the hospital for 6 days for intravenous rehydration. Some weeks later she was again admitted with vomiting and subsequent hypotonia requiring parenteral feeding. She was seemingly healthy until the age of 15 months when she was again admitted with similar symptoms. Thereafter, she experienced cyclic episodes of vomiting, hypotonia and constipation about 1-3 times per month (duration 5-12 days). The last 3 months, she had increasing pain during those episodes with ventricular retention, urine retention, need of parenteral feeding, morphine iv, antiemetics and sleep inducers. A treatment with propranolol (prophylactic against cyclic vomiting) was started, however, as no effect was noticed it was discontinued. From the age of 19 months, dystonic movements

(fingers, neck, arms) during those episodes with extended extremities (spastic like, but intermittent) were observed. Global developmental delay... Repeated abdominal ultrasound displayed normal findings except for ventricular/urine retention. Contrast X-ray of the oesophagus/stomach showed hiatal hernia with reflux. MRI brain (incl. brain spectroscopy), as well as several EEGs (incl 24hrs monitoring) were normal. Metabolic evaluation (urine/plasma/CSF), echocardiography and chest X-ray showed normal findings. Trio exome sequencing revealed a *de novo* heterozygous frameshift variant c.1146del, p.(Lys383Serfs*22) in *MSL3* (NM_078629.3).

Individual 3 – This female individual is the only daughter born to a non-consanguineous Caucasian couple. The family history was unremarkable except for her mother, who reported seizures in her first 2 years of life and a single episode when she was 15-year-old. The seizures had been attributed to difficult labour with forceps. Individual 3 was born at 40 weeks of gestation by spontaneous delivery after an uneventful pregnancy. At birth, weight was 2930 g (25th centile), length was 48 cm (25th centile) and occipitofrontal circumference (OFC) was 32.5 cm (10th centile), the Apgar scores were 10 at the 1st, and 10 at the 5th minute, and perinatal period was physiological. At 7 months of life several episodes of brief psychomotor arrest characterized by gaze palsy, hypertonus, clonic movements of the limbs, perioral cyanosis, and drooling were noted, so electroencephalography (EEG) was performed showing diffuse epileptiform anomalies while falling asleep. Antiepileptic treatment was then started with valproic acid with benefit. A brain MRI was also performed showing normal morphology. She underwent several additional diagnostic procedures (abdominal and cardiac ultrasound, eye evaluation) that provided normal results. Psychomotor development of the patient was characterized by hypotonia, motor clumsiness, and speech delay. At 26 months of age a Griffiths Scales test was performed showing a General Quotient (GQ) of 60. Clinical examination at 4 years at our facility only showed some aspecific dysmorphic features, a long and smooth philtrum and thin upper lip, and pes planus. Growth was regular, since weight, length, and OFC were 16.5 kg (25th centile), 98 cm (10th centile), and 49.2 cm ($10\text{-}25^{th}$ centile) respectively. Trio-based whole exome sequencing (WES) analysis was started, which detected the *de novo* heterozygous *MSL3* (NM_078629.3) missense variant c.1310A>C, p.(Asn437Thr).

Individual 4 - Individual 4 is a 5-year-old female with developmental delays, white matter abnormality on brain MRI, cerebral palsy and low muscle tone. She was born to unrelated healthy Caucasian parents after an uneventful pregnancy at 39 weeks of gestation. Acquisition of gross motor and fine motor skills were delayed. She sat at 14 months of age and walked independently at 19 months of age. First words were spoken by age on time. She receives occupational therapy and physiotherapy 30 minutes per week. She has normal vision and hearing. Trio exome sequencing

revealed the *de novo* heterozygous splice variant c.589-4_591del, p.? in *MSL3* (NM_078629.3) which was initially classified as variant of unknown significance and reclassified as pathogenic. In addition, a variant of uncertain significance (microduplication of 22q13.33) inherited from the healthy father was noticed. Mitochondrial DNA analysis was normal.

Individual 5 - Individual 5 is a female, born at 40 weeks of gestation to unrelated Caucasian parents. Family history is unremarkable. No prenatal complications were reported. She was born by C-section due to absent fetal movement. APGARs were reported normal. She weighed 3033 g, length was 50.8 cm, and head circumference was reported within normal limits. She passed newborn hearing screening and newborn screening resulted normal. Neonatal concern was failure to thrive for 3-4 weeks addressed with feeding therapy. She did not require a NIUC stay. Primary care doctor noted that her fontanelles were not closing and around 4 months old she was referred to Genetics due to short limbs, poor growth, difficulty feeding, and macrocephaly. Genetics work-up in the first year of life included microarray (resulted with 121kb deletion of 21q21.3, paternally inherited and classified as benign), skeletal dysplasia panel consisting of *COL1A1, COL1A2, COL2A1, COMP, SLC26A2, FGFR3, FLNA, HSPG2, SOX9, TRPV4* (resulted with two variants of uncertain significance in *HSPG2*; c.5755C>T was paternally inherited and c.8848G>A was maternally inherited), and methylation studies for Russell-Silver Syndrome (resulted normal). Metabolic work-up included normal oligosaccharide TLC and quantitative/qualitative for MPS. Around 1 year of age, a sleep study was performed and obstructive and central sleep apnea was noted. Additional sleep concerns include night terrors, snoring, delayed sleep latency and premature waking. Individual 5 has global developmental delays. She sat independently around 1 year, walked at 2 years, and said first words around 2 years. Right-sided sensorineural hearing loss was noted, and she now has hearing aids. She can now speak in complete sentences and follow one and two-step directions. She has nonsensical utterances. She has diagnoses of expressive language disorder and sensory integration disorder. She can walk independently but tends to lead with left leg and fatigues easily. She has ankle-foot orthoses (AFOs) and uses wheelchair for long distances. She falls frequently. She tends to favor left hand for fine motor skills. She requires assistance with dressing and using toilet. She has frequent staring spells which are suspected to be behavioral rather than seizures. She has had a normal EEG. Brain MRI at 3 years was unremarkable. She was diagnosed with autism around 4 years. She has trouble with swallowing and video swallow study noted delayed swallowing initiation and shallow vestibular penetration when swallowing liquids, but no aspiration was seen. She has gastritis, severe constipation, and reflux. She has dynamic muscle tone with periods of “floppiness”. She has spastic quadriplegic cerebral palsy. She has easy bruising, hyperhidrosis, and history of hemangioma on right shoulder which has resolved. Initial exome analysis was done around 4 years of age and showed the

heterozygous *de novo* frameshift variant c.1319dup, p.(Gly441Argfs*2) in *MSL3* (NM_078629.3) which then was classified as variant of uncertain significance. Reanalysis of exome was done at 5 years 7 months with reclassification of the variant in *MSL3* to pathogenic.

Individual 6

This 10-year-old female is the second of two children born to a non-consanguineous Caucasian couple with a history of ADHD in the father, the brother, the paternal aunt and the paternal uncle, as well as learning difficulties in the father and two paternal cousins. Pregnancy was marked by polyhydramnios noted at 7 months of pregnancy. She was vaginally delivered at term weighing 3402g. She nursed poorly. She failed newborn hearing screen and was diagnosed with bilateral moderate to severe sensorineural hearing impairment. She had no eye contact until about 3 months of age, smiled at 6 to 7 months, sat at 8 to 9 months and walked at 23 months. She was able to feed with spoon and fork at 4 years and started communicating with sign language. She has a short attention span. By the age of 8 years, she presented with increasingly aggressive and self-abusive behavior; she was hitting her head when upset and developed tics. At 10 years, functioning was at 3 to 4-year level and she uses bilateral hearing aids. She can recognize alphabet letters and numbers as well as sight words including names of family members, but she is not able to read. She is able to sign words and phrases, but has difficulty writing due to abnormal grasp of writing implements with stiff hands. She is unable to do maths. She walks with her knees bent and seems to have difficulties walking with occasional falls. She continues to have major behavioral outbursts, requiring treatment with chlorpromazine. She is toilet trained. Current medications include methylphenidat, clonazepam, clonidine, melatonin, and chlorpromazine. She had myringotomy tubes twice. Her vision was not formally tested but appears normal. In the primary dentition, the right lateral incisor was fused with the first bicuspid and both were small. She has frequent staring spells that are felt to be behavioral in nature with a normal EEG. She has no respiratory problems. Constipation and straining with urination are present. At 10 years, her height was 132.2cm (10th centile), weight 24.8 kg (3rd to 10th centile) and head circumference 51.8 cm (25th centile). Decreased facial expression and a deep hoarse voice were noticed. She has telecanthus with inner canthal distance at the 90th centile and outer canthal distance at 25th to 50th centile, bilateral epicanthal folds and squared nasal root. Full extraocular movements with normal pupillary responses and creases under the eyes. Ears low set, posteriorly rotated, and small (10th centile) with overfolded upper helices and anteverted earlobes. The philtrum seems to be poorly defined, and she has a small mouth with normal lips and a high narrow palate with intact uvula. Narrow chest with pectus carinatum and markedly underdeveloped pectoralis major muscles were observed. On examinations, a stiffness of movement of small joints of hands with limitation of extension were noticed. She has bilateral pes planus with hallux valgus, especially

on the right foot. Unusual posture with a tendency to keep arms extended with hands clasped and knees bent with hyperlordosis. Deep tendon reflexes were 3+ at the knees and 2+ elsewhere. No sequence variant or deletion involving GJB2 and GJB6 (connexin 26 and 30) genes was detected. A hereditary hearing loss panel showed a pathogenic homozygous deletion of the stereocilin (*STRC*) gene. No pathogenic variants in *BRAF, CBL, HRAS, KRAS, MAP2K1, MAP2K2, NRAS, PTPN11, RAF1, SHOC2, SOS1* (Noonan syndrome panel) were detected. Array CGH using a 180k oligoarray revealed no evidence of genomic imbalance. Single exome sequencing revealed the heterozygous frameshift variant c.808_809del, p.(Pro270Valfs*8) in *MSL3* (NM_078629.3) which was reported as a variant of uncertain significance and later reclassified as likely pathogenic. No parental studies were done.

Individual 7 – Individual 7 is a 16-year-old female who is the younger of two children and was born to healthy non-consanguineous parents of British descent. Her older brother as well as her three paternal half-siblings are healthy. The family history is negative for known genetic problems. Individual 7 was diagnosed with autism and developmental delay in early childhood. She has received special education and additional supports. She is not fully toilet trained at the age of 16 years. She can follow simple commands and has a communication device, but she only used it to continually ask for food. She has not had a seizure in years but continues on her medication. She has episodes of behavioral outbursts, with crying and hitting. Those episodes occur a few times a week and often only resolve with the administration of Lorazepam. She is prone to wander and can get lost. She likes to fiddle with something in her hands and likes to rock. Moreover, she enjoys music and being in the water. She is mildly dysmorphic with a high forehead, arched eyebrows, a down turned mouth and a round face. Height was 156.8 cm (17th centile), weight 69.4 kg (84th centile) and head circumference 56 cm (94th centile) at the last follow-up. Her fingers are tapered and her thumbs are proximally placed. Lungs were clear, heart sounds normal and abdomen was soft with no organomegaly. Trio exome sequencing revealed a heterozygous *de novo* deletion of exon 6-8 of *MSL3* (NM_078629.3) which was classified as pathogenic.

Individual 8 - This individual is a 17-year-old female, born to non-consanguineous Hispanic parents. Her family history is significant for an older brother with ADHD, a younger sister who has mild neurocognitive delay, and two female paternal cousins who were described as “slow.” Her mother was in a vehicular accident a week before delivery, but there was no apparent fetal distress. Individual 8 was born at 39 weeks gestational age via an induced vaginal delivery with a birth length of 48 cm (29th centile) and weight of 3.77 kg (85th centile). After birth, she underwent a day of phototherapy for jaundice. She was discharged from the hospital on her second day of life. In early infancy she was noted to have feeding problems and hypotonia. Her gross motor milestones were

reached within the upper limit of normal. Specifically, she was able to pull into a stand at 12 months of age and walk unassisted at 15 months of age. In contrast, her speech development was delayed. In her first year of life she only cooed. She was able to say “ma ma” and “da da” after she was a year old. She was putting two words together at 3 years of age and was speaking in short sentences at 4.5 years of age. Over time, she was diagnosed with autism spectrum disorder, intellectual disability, bradykinesia, parkinsonism, hypophonia and increased tone. She underwent bilateral lateral rectus recessions and currently has intermittent exotropia and wears glasses for myopia. An EEG and brain MRI performed at 14 years of age were normal. At 17 years 8 months of age her height was 148.5 cm (1st centile), weight 60.8 kg (69th centile), and OFC 58.8 cm (>98th centile). She has short stature, macrocephaly, telecanthus, extropia, and facial asymmetry. An array-based copy number variant (CNV) analysis preformed at Baylor Genetics revealed that she and her sister carry an Xp22.31 duplication (minimum chrX:6,866,449-8,115,153, maximum chrX:6,279,319-8,199,482; hg19) in the steroid sulfatase (*STS*) region. Although it remains uncertain whether recurrent duplications in this region alone are associated with an abnormal phenotype, a search of Baylor Genetics’ clinical database reveals that this gain is commonly inherited from a normal parent. Additional testing included urine organic acids and creatine-guanidinoacetate levels that were normal. Exome sequencing detected the heterozygous frameshift variant c.1168_1169del, p.(Lys390Glufs*6) in *MSL3* (NM_078629.3). This variant was not seen in her mother, but her father did not provide a sample for testing.

Individual 9 – This male individual is the only child born to non-consanguineous Caucasian parents. Both of his parents are alive and well. He was born at 38+4 weeks of gestation with a birth weight of 4.47 kg and a birth length of 52.1 cm. He has a history of developmental delay, hypotonia, and macrocephaly. His parents first noted developmental concerns at 4 months of age because he was not holding his head up. On his most recent assessment, his height was 88 cm (10th centile), weight was 13.2 kg (33rd centile), and head circumference was 54.5 cm (>98th centile). He has macrocephaly and plagiocephaly. His ears are small, and his nostrils flare out a bit. He has a broad forehead and short upturned nose. He holds his tongue outside of his mouth often. He is mildly coarse. He has stiff 5th fingers bilaterally, but no contractures. He has flat feet and low muscle tone. Furthermore, he previously had rectal prolapse. Trio exome sequencing revealed the *de novo* hemizygous nonsense variant c.913C>T, p.(Gln305*) in *MSL3*, classified as pathogenic.

Individual 10

Individual 10 is a 4-year-old male born at 39 weeks gestation to 41 year old, Caucasian, Non-Hispanic, non-consanguineous parents. A full older brother had no significant medical or cognitive concerns.

Delivery was by C-section at the time of spontaneous labor due to low transverse placental placement and a history of prior C-section. Non-invasive prenatal testing was normal. Birth parameters were within normal limits, (weight 3960 g, length 53.3 cm, head circumference 35.6 cm) and APGARS were 8 at 1 minute, 9 at 5 minutes. On newborn exam a heart murmur, umbilical hernia, bilateral hydroceles, and left lacrimal duct stenosis were noted. He was discharged to home on day three of life. The parents had concerns about his eye contact and social engagement at 2 months. As an infant, he was diagnosed with anisocoria and monitored for positional plagiocephaly (did not require helmet therapy). He was referred for developmental assessment by his pediatrician after concerning responses on his Ages & Stages Questionnaire at his 9 month well child checkup. Developmentally he had global delay, with less significant motor delay as compared to his language and social domains. He rolled and sat independently for brief periods at 11 months. By 1 year he had a well-developed pincer grasp. He first walked independently at 2 years, 3 months. At 16 months he had bilateral myringotomy tube placement and bilateral surgery to treat intermittent alternating exotropia. At 17 months he was first examined by a medical geneticist. On physical examination he had relative macrocephaly, low posterior hairline, prominent nasal bridge with bulbous tip, low set, posteriorly rotated prominent ears with overfolded helicies, and slightly tapered fingers. Molecular evaluation included negative *FMR1* evaluation for trinucleotide repeat expansion (23 repeats observed) and a normal male result on chromosome microarray analysis: Arr(1-22)x2,(XY)x1. A brainstem auditory evoked response evaluation of hearing shortly at this time noted possible low frequency hearing loss bilaterally, but hearing was deemed adequate for speech acquisition. At 18 months he was admitted to a local hospital with increased fussiness, change in mental status and decrease oral intake. An MRI of the head was negative and demonstrated normal morphology. Abdominal ultrasound revealed an illeocolic intussusception which required laparoscopic reduction. He has had two additional hospital admissions for influenza and pneumonia. At 3 years, he was evaluated in the Duke Genome Sequencing Clinic as a participant in a research study, (Duke Protocol 00032301) where trio-based exome sequencing (ES) analysis was completed. Laboratory testing detected a *de novo* hemizygous *MSL3* (NM_078629.3) stop gain variant c.11105C>T, p.(Gln369*). Developmental assessment determined that he met DSM-V criteria for Autism Spectrum Disorder. Performed due to periods of behavioral arrest, a sleep deprived EEG during this period was normal in both awake and asleep states. At age 4 years, he is functionally non-verbal, but does have a few signs, follows single step instructions and responds to his name. He continues to be well grown with normal growth parameters at 3 years and 5 months. (weight: 17.3 kg, 89%, height 101 cm, 73%, head circumference: 52.5 cm, 90th %) and 4 year 1 month (weight 18.6 kg, 82%, height 104.1 cm, 59%). Funding for the Duke Genome Sequencing Clinic was provided by Duke University Health System.

Individual 11 - This male individual is the first child of non-consanguineous healthy Chinese parents. During pregnancy reduced fetal movements, oligohydramnios and cleft lip and suspected cleft were detected. No other structural abnormalities were present. He was born at term by emergency caesarean section due to fetal distress. Birth weight, length and OFC were 3380g (29th centile), 53cm (59th centile) and 35cm (32nd centile). Incomplete left cleft lip was noted, but palate was intact. He had two pre-auricular tags on the right and his right external ear had a prominent serpiginous antihelix stem and a mild cleft. Global delay was first noticed at 14 months of age (unaided sitting at 7-8 months, unsupported standing for a few seconds around 24 months). By 18 months of age, he was diagnosed with autism spectrum disorder. At last follow-up at $4\frac{2}{12}$ years he walked unsupported. So far, he has no expressive language. Intradural lipoma C6/7, but no other abnormalities were evident on cMRI. Cardiac and abdominal ultrasounds revealed normal results. Unilateral (right side) sensorineural hearing loss was present and he suffered from recurrent otitis media (grommets in situ). He had longstanding vomiting, possibly secondary due to esophageal sphincter dysfunction. The patient developed postnatal short stature and his anthropometric data at $3\frac{10}{12}$ years were: height 93cm (1 centile, -2.29 SD) and weight 15kg (25 centile, -0.69 SD). Fragile X testing was negative, and microarray showed two interstitial microduplications involving chromosome region Xp22.33 or Yp11.22. The assay was unable to determine if the duplications are within the Xp or Yp region due to shared PAR1 homology. The duplication contains the whole of *SHOX* gene. Further parental analysis was not undertaken. The parents returned for preconceptual counselling when the patient was 2 years of age. Singleton WES was undertaken and the hemizygous splice-site variant c.1382-1G>A in *MSL3* (NM_078629.3) was detected. Segregation study (Sanger) suggested that his phenotypically normal mother is mosaic for the *MSL3* variant. The mutation load of approximately 8% was estimated using ddPCR. The variant was classified as likely pathogenic.

Individual 12 - This male individual is the youngest of three children to a non-consanguineous Caucasian couple and was born at 39 weeks of gestation, following an uneventful pregnancy. Birth weight (3530g; 56th centile), length (49.5 cm; 16th centile) and OFC (35.5 cm; 58th centile) were normal. Hypotonia was noted at birth as well as feeding issues. High arched palate, thin upper lip and extra hair whorl in front were evident. Family initially presented to neurology at 10-11 months of age. At that time, it was due to lack of crawling, very limited upper body strength, excessive drooling and failure to take solid foods. Fine motor skills were underdeveloped. Sitting, standing and walking without support were possible at 9-12 months, 12 and 23 months, respectively. He had very little speech before he started speech therapy at 2 years. At 3 years he could speak 2-3 words in combination. Around 2.5-3 years after starting speech therapy, he increased to more defined words and at 3-4 years he spoke sentences. Neuropsychological examination at 6 years showed an overall

intellect within the borderline range with distinct strength in language comprehension (WISC-V: FSIQ=72, VCI=98, VSI=67, FRI=74, WMI=76, PSI=53). Moreover, autistic spectrum disorder and a generalized anxiety disorder with OCD features were diagnosed. Brain MRI showed underdeveloped pituitary. His auditory function was normal. The results of abdominal and cardiac ultrasound were inconspicuous. Esotropia, hyperopia, constipation and mild to moderate muscle weakness were present. Anthropometric data were normal at $3\frac{11}{12}$ years (height 98 cm, 17th centile; weight 13.9 kg, 9th centile; OFC 52 cm, 89th centile). Microarray was normal as was SMA (Spinal Muscular Atrophy) testing and mucopolysaccharide screen. Muscle biopsy was nondiagnostic. Trio exome sequencing was undertaken and was initially reported as normal. Reanalysis done one year later identified the *de novo* hemizygous frameshift variant c.1125_1141dup17, p.(Met381Argfs*30) in *MSL3* (NM_078629.3).

Individual 13 - Individual 13 is a 6 year 3 month old boy who was born at 40 weeks gestation via Cesarean section for failure to progress to his then 28-year-old mother and 29-year-old father. The pregnancy was complicated by polyhydramnios, with prenatal ultrasound findings of short appearing limbs and macrocephaly. His birth weight was 3.95 kg (90th centile), and there were no perinatal complications. He had poor weight gain in the first few weeks after birth and was treated for gastroesophageal reflux disease (GERD) with some improvement in weight. Due to his macrocephaly he had a head ultrasound at 7-months and a brain MRI at 13-months that were both normal. He had delayed milestones, rolling at 9 months, sitting at 12 months, and walking at 2 years of age. His first words were at 3 years of age and he started speaking in simple sentences around 4 years of age. He has had normal audiology evaluations. Initially he made poor eye contact and did not seek out interaction with parents or others, and he was diagnosed with autism spectrum disorder at 5 years of age. His social skills have improved over time, but he prefers to play by himself. He has a short attention span and is prone to anxiety when in crowds. He is not aggressive and does not exhibit self-harming behaviors. He has trouble sleeping at night, which has been helped somewhat by melatonin and physical activity during the day. He has hypotonia with frequent drooling, though sometimes will feel stiff, especially when getting picked up. He used to get sick a lot as a younger child but now seems to have a normal immune system, though he does have chronically loose stools. He receives physical, occupational and speech therapies and is in a special education classroom setting. At 6 years 3 month his weight was at the 87th centile, height at the 66th centile, and head circumference greater than the 98th centile, with a Z-score of +3.9. He has dolichocephaly with frontal bossing, and thick, coarse, curly hair with a frontal upsweep. His ears are posteriorly rotated with slightly dysplastic helices, and he has down-slanting palpebral fissures. He has a prominent nasal bridge and mild retrognathia. He has a large, faint nevus flammeus on his forehead, and a smaller one at the

nape of his neck. There is also a large, irregularly shaped hyperpigmented macule in his right groin. A chromosome microarray identified two variants of uncertain clinical significance: a 284 kb copy number gain at 6q27, including the *MLLT4* and *KIF25* genes, and a 107 kb copy number loss at 15q15.3, including the *STRC, CATSPER2*, and *CKMT1A* genes. Both copy number variants were inherited from his healthy and developmentally normal father. Biochemical evaluation included normal plasma amino acids, plasma acylcarnitine profile, ammonia, lactic acid and thyroid stimulating hormone. Whole exome sequencing (WES), performed as a trio, identified the *de novo* hemizygous missense variant c.1370T>C, p.(Leu457Pro) variant in *MSL3* (NM_078629.3), which was initially classified as variant of uncertain significance and reclassified as likely pathogenic. There were no other reported variants of interest on the WES.

Individual 14 - This is a 3-year-old boy born to healthy unrelated parents**,** who has a healthy older 5-year-old brother. He was born weighing 1.3kg at 40 weeks by emergency caesarian section for fetal distress. OFC was on the 70th centile. Delayed development and central hypotonia was noted at 3 months of age. He had recurrent respiratory infections requiring hospitalization. At 3 years of age, he is pulling to stand, is able to grab items, and to feed himself. He has no speech and is not yet pointing. He has normal growth parameters, has a moderate pectus carinatum, with no dysmorphic features. His metabolic studies, microarray, fragile x and prader willi and MRI brain are all normal. Whole exome sequencing showed a *de novo* hemizygous pathogenic variant in *MSL3* (NM_078629.3): c.590_593del, p.(Leu197*).

Individual 15 - This male individual was born at term with a history of an uncomplicated pregnancy and delivery to healthy unrelated Caucasian parents. His birth weight was 3.81 kg and his birth length was 52.1 cm. His neonatal course was likewise unremarkable. The family history was negative for other individuals with developmental problems or congenital anomalies. His early developmental milestones were achieved at a late-normal time. A persistent central (supranuclear) hypotonia was noted at an early age. He was evaluated at 3 years of age and was noted to have global delays. His speech pattern showed no words but good receptive language. He was reported to fall frequently due to low tone and discoordination. On examination he had a few remarkable craniofacial features including macrocephaly (FOC >>98th centile), frontal bossing, and somewhat coarse features. Below the neck, he was moderately overweight and had tapering fingers. No other anomalies were identified. Medically he was found to have hydronephrosis and dilated ureters. He had a dilated aortic root and a patent foramen ovale. Diagnostic studies include normal CK level, thyroid functions and metabolic screening. A brain MRI identified a supra-sellar arachnoid cyst and was otherwise

reported as normal. Trio exome sequencing identified the *de novo* hemizygous in-frame deletion c.1362_1364del, p.(Gln454del) in *MSL3* (NM_078629.3).

Individual 16 - This 9-year old boy was the second child of a non-consanguineous couple of Middle-Eastern origin. His older sister was healthy and the family history unremarkable. He was born via cesarean section at 36 weeks following a pregnancy with polyhydramnion and a pyelo-ureteral junction stenosis (PUJ) on the left side. At birth, his weight was 3750 g, his length 49.5 cm and his head circumference 35.5 cm. Because of the PUJ, he stayed at the neonatology unit for 9 days. He spontaneously developed diuresis, but the hydronephrosis remained until the PUJ was surgically corrected at the age of 3 months. During the first two years of life he had recurrent upper respiratory tract infections and several episodes of bronchiolitis for which he received inhalants with ipratropium bromide, salbutamol and budesonide. Allergy for egg white proteins was noted. His neurodevelopment was delayed. He sat independently at the age of 12-13 months and walking at 30 months. He spoke his first words at 23 months and could speak in short sentences at the age of 3 years. Physiotherapy was started at 9 months. He received speech treatment, and ergotherapy from the age of 2.5 years. Later-on he showed characteristics of an autism spectrum disorder. He had an instable bladder and has not acquire urinary continence yet at the age of 9 years. Further work-up at the age of 13 months included normal results for urinary organic acids, oligosaccharides, mucopolysaccharides, and sialic acid (116 µmol/mmol creatinine (ref 0-74)). Abdominal ultrasound showed remaining bilateral pyelectasis. Brain ultrasound showed dilated ventricles, which remained stable over time. Brain MRI further showed mild external hydrocephaly, and prominent Virchow-Robin spaces. A skeletal survey at the age of 12 months showed delayed skeletal maturation, and a low mineralized skeleton, and a prominent J-shaped broad aspect of the sella turcica. Molecular karyotyping showed two inherited variants: Hg19 arr 5q21.1q21.1(99760791-99930438)x1 mat, arr 11q14.1q14.1(81540019-81620208)x1 pat. A panel for RASopathy genes (*PTPN11, SOS1, BRAF, MEK1, MEK2, KRAS, HRAS*), *FGFR3, PTEN* and *COL1A1* and *COL1A2* were normal. At the age of 4 years and 4 months, he was reevaluated because of a focal and lateralized seizure for which carbamazepine was started. A CT-scan of the brain as well as an electroencephalogram was normal. His head circumference was 54.8 cm (+2.7 SD), his weight 17.5 kg (p50), and his length 100.8 cm (p3) and showed increased deep tendon reflexes at the lower limbs. At the age of 9 years, his weight was 26.9 kg (p25-50), his length 127.3 cm (p3) and his head circumference 55.8 cm (+2.8 SD). His arm span was 119.5 cm with mainly a rhizomelic component. He had coarse facial features, with a low anterior hair line, mild hypertelorism, small epicanthal folds, protruding ears with thick helices and downturned corners of the mouth. His tongue has some deep creases, and he had hypertrophic gums. He showed generalized hirsutism on arms, back and forehead. His thorax appeared relatively

small, and his liver extended 2 cm below the ribs, likely due to the smaller thorax. He had bulging of the soft tissue on the pubis, a large scrotum, but normal testes. He showed slight contractures in the knees but not in the elbows. His legs were relatively stiff and he had increased deep tendon reflexes with some repetition and extension of the reflectory zone, but a normal plantar reflex. Trio exome sequencing with analysis of a panel of 1150 intellectual disability/epilepsy related genes showed a *de novo* hemizygous variant (c.1171+2_1171+4del; p.?) in *MSL3* (NM_078629.4).

Individual 17 - This 19-year-old male individual was born at 40 weeks of gestation following an uneventful pregnancy. Family history is unremarkable. He had global developmental delay. Currently, he is nonverbal has quadriplegic cerebral palsy, autism spectrum disorder, slow GI transit, Pelger Huet anomaly, high myopia/astigmatism, airway clearance impairment and chronic insomnia. MRI showed leukodystrophy. He requires a wheelchair, a GI tube, and catheterization. He had a left branchial cleft remnant removed and has abnormal long bone trabeculation on x-ray. Physical examination showed tapered fingers, right periauricular tag, relative macrocephaly, and limitations of pronation/supination suggestive of radioulnar synostosis. Previous genetic testing has been inconclusive (361kb duplication on chromosome 10p15.3, negative testing for Fragile X syndrome, Coffin-Lowry, Familial Dysautonomia, Branchio-oto-renal syndrome, normal metabolic studies). Trio exome sequencing showed a *de novo* hemizygous nonsense variant (c.961C>T, p.(Gln321*)) in *MSL3* (NM_078629.3) which originally was classified as a VUS but recently was reclassified to pathogenic.

Individual 18 – This male individual is a product of his mother's second pregnancy of five. The maternal age was 22 and the paternal age was 24 at the time of the pregnancy. At five months gestation, the mother had food poisoning and required IV fluids and promethazine, and also terbutaline for contractions. No other complications occurred during pregnancy. The spontaneous vaginal birth occurred at 37.5 weeks' gestation without complications. His birth weight was 2.6 kg and he did not have any dysmorphism noticed as an infant. He had significant vomiting from birth that was evaluated when it became bloody and he was found to have ulcers. He was also found to have a hiatal hernia. For these reasons he had a Nissen fundoplication. His GI issues resolved after that. This individual was last evaluated at 19 years of age and has a history of global developmental delay and episodes of encephalopathy with regression. He has macrocephaly, low hairline, and has some thickening of his facial features and also possibly some of his joints. His early development was not concerning until 2-3 years of age when he was noted to be falling behind his peers. He crawled at 6 months and walked at 10 months. Around the age of 2 years, he fine motor delays and speech/language delays became apparent, and he was still having vomiting and recurrent sinus infections and tonsillitis. He had vision problems and was diagnosed as being extremely near-sighted,

and he was given glasses. He was found to have both conductive and sensorineural hearing loss in both ears. These were initially treated with PE tubes, which did not help. He received hearing aids at age 5, and his speech and language skills improved to some degree after this, although he still had delays in language. His fine motor skills continued to be delayed. He progressed through grade school and high school with an individualized educational plan in place throughout his entire schooling. He graduated from high school last year. His IQ post his 13-year-old episode was 55, he reads at about a grade 5 or 6 level and performs math at a similar grade level. He has had 2 episodes of developmental regression, occurring in association with acute encephalopathy, occurring at ages 13 and 18. He apparently recovered, partially, from the episode at age 13 over a period of 6 months. It took him 6 months to recover from the episode at age 18, but again only a partial recovery. This individual has a history of involuntary movements. They consist primarily of a left shoulder twitch. He also has some stuttering and some garbling of his speech. He has a tremor with reaching, fine motor skills, or action, but not at rest. There is no dystonia or chorea. He has never had seizures. The family has noticed coarsening of his facial features since age 13. He seems to have developed hairiness of the body more than they might have expected based on other family members. He also has required multiple surgeries for hammer toes. On neurological review of systems, this individual has moderate conductive and sensorineural hearing loss bilaterally and requires hearing aids. He has had migraines since age 13. Since age 13, they have noticed spasticity in the legs, and he is on baclofen for this. He has also had increased deep tendon reflexes and clonus. He has a high pain tolerance but normal sensation. Clinical testing has included MR Spectroscopy showing a relatively low NAA with a possible lactate peak. MRI brain showed nonspecific T2 hyperintensities in the white matter but no evidence of a leukodystrophy. EEGs have shown a slow background with no epileptiform activity. Biochemical evaluations for lysosomal, peroxisomal, inborn errors of metabolism, creatine disorders, CDG were unrevealing. He had an infectious workup in the CSF which was negative at the time of his encephalopathic episodes. His autoimmune workup has also been negative. Testing for lysosomal storage disorders including urine mucopolysaccharides and oligosaccharides and oligosaccharides in the white blood cells was unrevealing. He has had a negative carbohydrate-deficient transferrin test. Oxysterols were normal ruling out Niemann-Pick C. Neurotransmitters raised the possibility of tetrahydrobiopterin metabolism defect based on low concentrations of tetrahydrobiopterin, homovanillic acid and 5-OH-indolacetic acid, but GCH1 gene encoding GTP cyclohydrolase was normal. Unrevealing genetic testing included normal chromosomal microarray, fragile X, an Intellectual disability/developmental delay next-generation sequencing panel, and mitochondrial DNA sequencing. The proband had trio exome sequencing revealing a *de novo* hemizygous missense variant (c.1373G>T, p.(Arg458Leu)) in *MSL3* (NM_078629.3).

Individual 19 – Individual 19 is a 23-year-old man born at term following an uneventful pregnancy. He presented with global developmental delay and hypotonia evolving into intellectual disability, speech disorder, and ataxic gait disturbances. A hyperkinetic disorder became evident soon after birth, predominated by generalized dystonia. A brain MRI scan documented no gross neuroanatomical changes. He was enrolled in our large-scale WES project focusing on the etiologic origins of dystonia and analysis was performed using a trio design. The *de novo* hemizygous nonsense variant c.1314C>A, p.(Tyr438*) in *MSL3* (NM_078629.3) was detected and classified as pathogenic. Genotype and phenotype information of this individual have recently been published in a large exome study on movement disorder dystonia.[7]

Individual 20 - The male individual was born to parents of Serbian descent. The paternal age was 34 years. There is little information about the mother, who died at the age of 62 years because of cancer. He has one sister who is 41 years old. The patient was born at 42nd week of gestation by normal delivery. At birth his OFC was 37 cm, length was 50 cm and weight was 3520 g. 10 minutes post partum he developed cyanosis and signs of infection. Postnatally a distended abdomen, club feet and dysmorphic features (face, ears) were noted. At the age of 4 months x-ray of the wrist joint showed some degree of decalcification. At the age of 6 months a biopsy of the colon/rectum revealed a neuronal intestinal dysplasia type B. At the age of 9 months and again at the age of 2 years he had a fracture of the femur. He could sit independently at the age of 1.5 years and walk at the age of 3 years. Speech development was difficult to find out in detail retrospectively: at the age of 7 years it is mentioned in a medical report, that he could speak single words. His spontaneous motor activities were reduced and a myopathic and dystrophic appearance is documented in medical reports from the age of 7 years. From the 11th year of life celiac disease and iron deficiency anemia are documented. At the age of 14 years a hemifundoplication was performed because of gastroesophageal reflux. At the age of 30 years he was seen as surgical outpatient because of dysphagia of unknown origin. At the last physical examination at the age of 30 years his OFC was 56 cm, length 166 cm and weight was 34.8 kg. The thoracic kyphosis was striking with sloping shoulders and a very meagre habitus. During the examination he was cooperative, quiet and friendly. As a child he attended special schooling. At the age of 30 years he lives now in the household of his sister and attends a sheltered workshop several hours a week. He can speak in short sentences. His movements are slow, he needs help to dress and undress, however can walk without assistance. Exome sequencing revealed a 4 bp deletion affecting the splice-site and predicted to result in a premature termination signal in *MSL3* (NM_078629.3) c.590_593del, (p.Leu197*). The variant was not present in his sister. The mother was not available for segregation analyses.[6]

Individual 21 - Individual 21 is an 11-year-old male who was the 3345 g product of a 40 week gestation, born via cesarean section to a then 30 year old (gravida 2, para 1-2) mother with early prenatal care. Pregnancy complications included identification of cysts on the umbilical cord. The mother was followed closely throughout the pregnancy. Prenatal testing included an amniocentesis for which the results were reportedly normal. Prenatal exposure and acute maternal illness during pregnancy was denied. Complications in the perinatal period include a NICU stay for five days due to apneic episodes and congenital contractures. He was discharged home on oxygen therapy and struggled with feeding from birth. He first sat at 2 years of age and walked at 3 years of age. He said his first word with meaning at 3 years of age and put 2-3 words together at 4 years. Medical history is significant for limb contractures, atrial septal defect s/p closure, bilateral mild conductive hearing loss, dysphagia, kyphosis, and global developmental delays. Currently, he uses complex speech, and will have back and forth conversations. He will build on things other people say, and picks up things that other people are interested in. His articulation is challenging; his mother understands most of what he says, his therapists and other people have said they understand around 30% of what he says. He has an augmentative communication device, which he will use it at speech and therapies but does not use it much at home because his family understands him. Genetic testing has included a Noonan Syndrome panel, a Distal Arthrogryposis Panel, and Chromosomal Microarray which were all normal. A broad neurodevelopmental panel identified a *de novo* hemizygous pathogenic variant in *MSL3* (NM_078629.3): c.865A>T, p.(Lys289*).

Individual 22 – Individual 22 is a 15-month-old male who was born at 37-weeks via vaginal delivery to 30-year-old mother. On ultrasound, mother was told there were “signs of dwarfism”. Patient had nuchal cord at delivery, otherwise there were no complications. Growth parameters at birth were within normal limits. At 15 months, he was able to sit up with assistance. Medical history is significant for failure to thrive, relative macrocephaly, hypotonia, developmental delay, laryngomalacia, sacral cleft, protuberant abdomen, abnormal fat pads, neonatal hyperbilirubinemia, and undescended testes. Brain MRI showed mildly enlarged ventricular system with transependymal flow and was otherwise normal. Individual 22 previously had a normal male SNP microarray. Whole exome sequencing identified a *de novo* hemizygous nonsense variant (c.1347C>A, p.(Tyr449*)) in *MSL3* (NM_078629.3) classified as pathogenic.

Individual 23 – Individual 23 is a 9.75 year old female who was born at term via Cesarean section after an uneventful pregnancy. As an infant, she had feeding difficulties as well as constipation. Developmentally, she was delayed, walking at 17 months and speaking at 29 months. She was diagnosed with mild bilateral sensorineural hearing loss. Neuropsychiatric testing revealed ADHD,

social pragmatic communication disorder, and generalized anxiety disorder. She had an EEG for staring episodes and repetitive blinking, and this demonstrated right-sided focal slowing during hyperventilation as well as eyelid closure blepharoclonus. A brain MRI at age 8 revealed non-specific T2 hyper intense foci in the bifrontal white mater. At her most recent outpatient visit, spasticity was noted at her knees bilaterally. She is mildly dysmorphic with diffuse hypotonia and an abnormal gait. Trio exome sequencing identified a *de novo* heterozygous pathogenic variant (c.566dup, p.(Tyr189*)) in *MSL3* (NM_078629.3). The individual also harbors the heterozygous nonsense variant c.3496C>T, p.(Arg1166*) in *MYO6* (NM_004999.3) inherited from her mother who has sensorineural hearing loss.

Individual 24 – Individual 24 was born at 38 4/7 weeks after a pregnancy complicated by fetal ultrasound findings suggestive of short limbs, choroid plexus cyst, and hydronephrosis, of which the latter resolved. Amniocentesis was performed and karyotype and microarray were normal. At birth he was appropriately grown with a weigh of 3.6 kg, and length of 52.1 cm but had relative macrocephaly with a head circumference of 35.6 cm and was noted to have macroglossia. He did well neonatally with a brief period of transient tachypnea. At 2 months of age he was seen by the genetics service nearest to his hospital of birth. He was making normal developmental progress and growing well. Noted were a large anterior fontanelle (9x9 cm), coarse facial features, epicanthic folds, underrotated ears, broad nasal bridge, mild retrognathia, enlarged protuberant tongue, small umbilical hernia, mild rhizomelic limb shortening, no skeletal asymmetry, and capillary malformations over the glabella and occiput. Methylation testing for Beckwith-Wiedemann syndrome, very long chain fatty acids, urine mucopolysaccharide screen, radiographic skeletal survey, and abdominal ultrasound were all normal. In follow up, further testing included molecular genetic testing for metabolic disorders, which also yielded normal results. After moving to another state, he was seen by a second genetics service at just under 2 years of age. By that time, he was demonstrating global developmental delays, episodes concerning for seizures but normal EEG, and had been found to have first and second degree A-V block. Relative macrocephaly and macroglossia persisted. Whole exome sequencing was performed and revealed a *de novo* hemizygous pathogenic variant in *MSL3* (NM_078629.3:c.973_974del, p.(Gln326Alafs*5)).

Individual 25 – Individual 25 is the second child of two children born to unrelated parents. She was born at term by spontaneous vaginal delivery weighing 3500g. A large head circumference was noted at birth. She sat at 8 months and walked at almost 2 years of age but her parents had concerns about her development from the age of a year. She was hospitalized at 6 months and 1 year for treatment of lower respiratory tract infections. Physiotherapy assessment noted low muscle tone. She has a

speech and language delay with only a few single words at 2 years. Her voice sounded a bit hoarse with a nasal tone. At 3 years and 10 months she could follow single staged commands and there were concerns about her understanding. Her head circumference was 53.7cm (3.185 SD). Weight and height at 3 years and 6 months were 15.36kg (0.180 SD) and 95.8cm (-0.803 SD) respectively. On examination features included overfolded helices, beaked nose with broad nasal bridge, tapering of the fingers, narrow chest, short neck, flat feet and slim calf muscles. On review at 13 years and 5 months she was attending a special educational needs school and had a diagnosis of dyspraxia and poor motor planning, autistic spectrum disorder and a motor tic causing tilting of the head. Weight was 46.68 kg (0.099 SD) and height 157.3cm (0.001 SD).

Investigations included normal female karyotype, array CGH, FRAX, Prader-Willi methylation studies, baseline biochemical investigations and MRI brain which revealed no abnormality. She was recruited into the 100,000 genomes project under Genomics England for trio whole genome sequencing revealing a *de novo* heterozygous nonsense variant in *MSL3* (NM_078629.4:c.1372C>T, p.(Arg458*)) which was classified as pathogenic.

Supplementary Figures

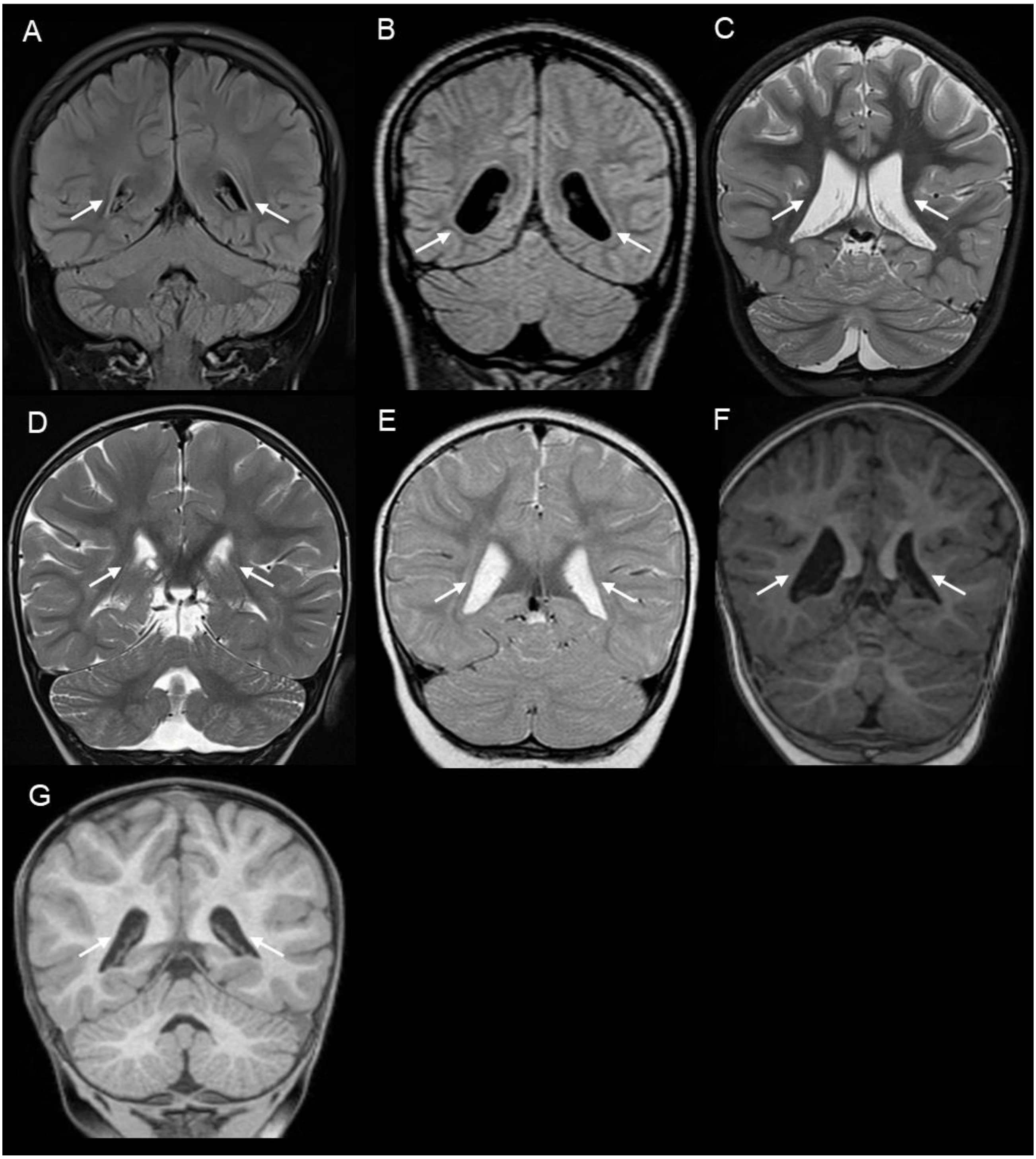

Figure S1: Brain MRI findings in individuals with pathogenic variants in *MSL3*: A) Brain MRI image (FLAIR, coronal view) of a healthy individual at the age of 13 years. The white arrow indicates the trigonum of the lateral ventricles B) –G) Brain MRI images (coronal views) of individuals with variants in *MSL3* (FLAIR sequence [B)]; T2-weighted sequence [C)-D)], T1-

weigthed sequence [E)-G)]). B) Individual 17 (age 13 years). C) Individual 16 (age 5 years). D) Individual 11 (age 24). E) Individual 3 (age 14 months). F) Individual 1 (age 12 months). G) Individual 2 (age 20 months). In individuals with pathogenic variants in *MSL3* a variable dilatation at the trigonum of the lateral ventricles was observed (white arrows; please compare with normal findings in panel A).

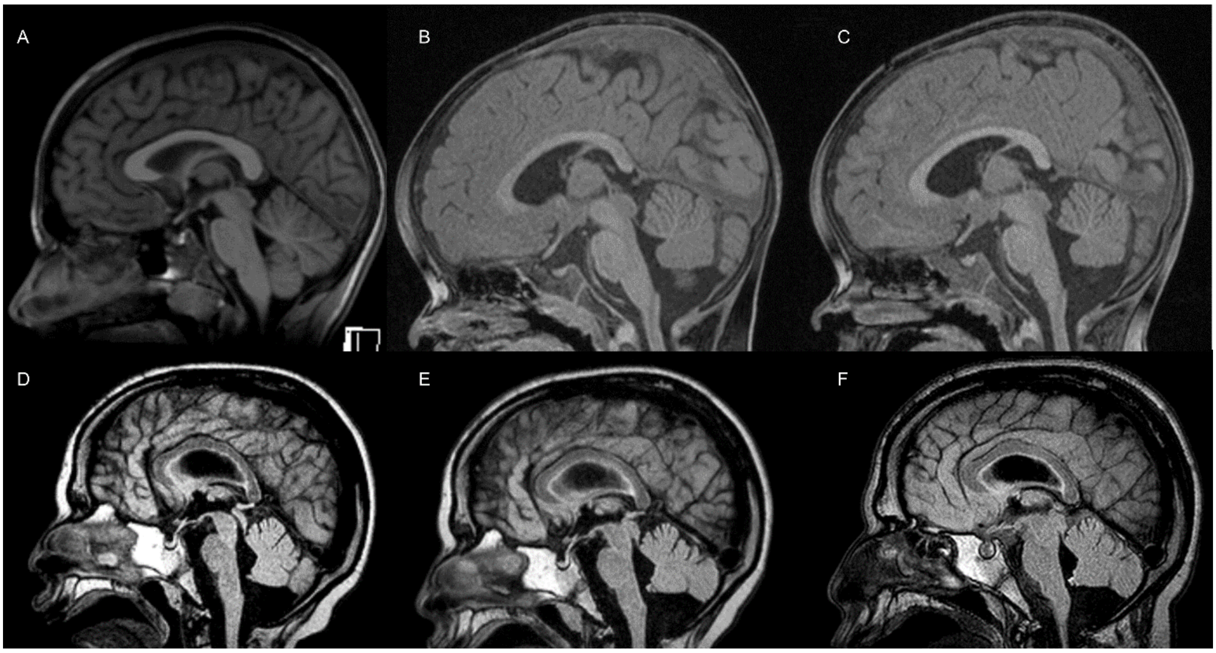

Figure S2: Longitudinal brain MRI findings in individuals 2 and 17: A) Brain MRI image (FLAIR, coronal view) of a healthy individual at the age of 13 years. B) –C) Brain MRI images (sagittal views) of individual 2 (T1 sequence); and D)-F) of individual 17 (FLAIR sequence). B) Individual 2 at age 10 months and C) at age 20 months. D) Individual 17 at age 13 years, E) at age 13½ years and F) at age 17 years. In individuals 2 and 17, no progression of cerebellar abnormalities could be observed.

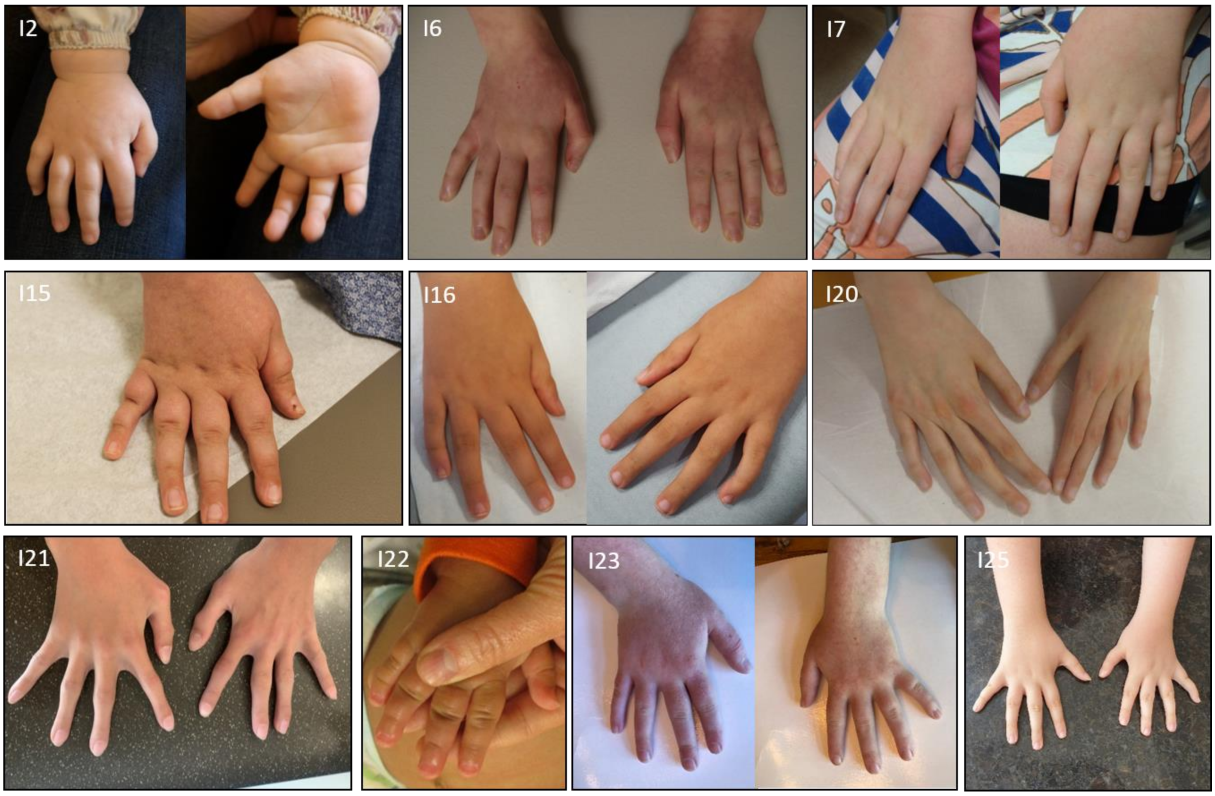

Figure S3: Pictures of the hands of individuals with pathogenic variants in *MSL3*. Minor abnormalities of the digits (e.g. tapered fingers, clinodactyly, long and slender fingers) are present.

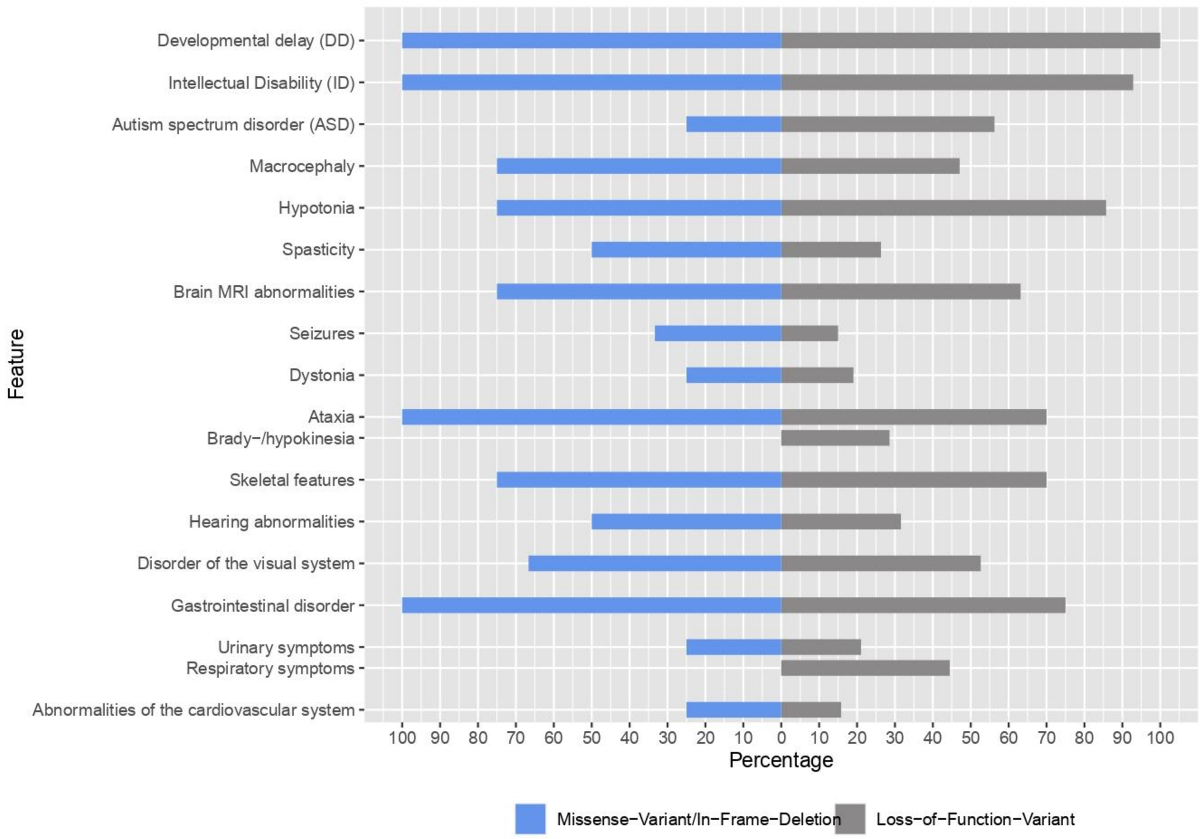

Figure S4: Variant specific differences in individuals with pathogenic variants in *MSL3*. Back-to-back chart visualizes the frequency of clinical features (in percent) present in individuals with missense variants/in-frame deletions (blue bars, n = 4) and individuals with loss-of-function variants (grey bars, n = 21). The distribution of no feature was significantly different between the respective subgroups (Fisher's exact test).

Supplementary Tables

Table S1: Extensive clinical data of *MSL3* cohort.

Table S2: List of *MSL3* variants identified in this cohort including ACMG criteria.

Individual	Type of variant	Nucleotide alteration (NM_078629.4)	Protein alteration (NP_523353.2)	Genomic position hg19 (NC_000023.10)	Zygosity	Exon number	Inheritance	CADD score[8]	PhyloP score[9]	ACMG	ACMG criteria	gnomAD
1	Spl	c.1466+1G>A	p.?	g.11790825G>A	het	12	*de novo*	33	-	P	PVS1_strong PS2 PM2 PP3	not present
2	FS	c.1146del	p.(Lys383Serfs*22)	g.11783823del	het	9	*de novo*	25.3	-	P	PVS1 PS2 PM2 PP3	not present
3	MS	c.1310A>C	p.(Asn437Thr)	g.11790303A>C	het	11	*de novo*	20.7	2.51	LP	PS2 PM1 PM2 PP3 BP1	not present
4	Spl	c.589-4_591del	?	g.11780952_11780958del	het	7	*de novo*	-	-	P	PVS1 PS2 PM2	not present
5	FS	c.1319dup	p.(Gly441Argfs*2)	g.11790312dup	het	11	*de novo*	24.8	-	P	PVS1 PS2 PM2 PP3	not present
6	FS	c.808_809del	p.(Pro270Valfs*8)	g.11781957_11781958del	het	8	unknown	32	-	P	PVS1 PM2 PP3	not present
7	Multi-Exon deletion	c.(465+1_466-1)_(908+1_909-1)del	?	g.(11779702_11780248)_(11782058_11783585)del	het	6-8	*de novo*	-	-	P	PVS1 PS2 PM2	not present
8	FS	c.1168_1169del	p.(Lys390Glufs*6)	g.11783845_11783846del	het	9	*de novo*	33	-	P	PVS1 PM2 PP3	not present
9	NS	c.913C>T	p.(Gln305*)	g.11783590C>T	hem	9	*de novo*	38	-	P	PVS1 PS2 PM2 PP3	not present
10	NS	c.1105C>T	p.(Gln369*)	g.11783782C>T	hem	9	*de novo*	39	-	P	PVS1 PS2 PM2 PP3	not present
11	Spl	c.1382-1G>A	p.?	g.11790739G>A	hem	12	maternal (8% mosaic in mother)	35	-	P	PVS1_strong PM2 PP3	not present

12	FS	c.1125_1141dup17	p.(Met381Argfs*30)	g.11783802_11783818dup17	hem	9	*de novo*	28.3	-	P	PVS1 PS2 PM2 PP3	not present
13	MS	c.1370T>C	p.(Leu457Pro)	g.11790363T>C	hem	11	*de novo*	25	3.839	LP	PS2 PM1 PM2 PP3 BP1	not present
14	NS	c.590_593del	p.(Leu197*)	g.11780957_11780960del	hem	7	*de novo*	32	-	P	PVS1 PS2 PM2 PP3	not present
15	InDel	c.1362_1364del	p.(Gln454del)	g.11790355_11790357del	hem	11	*de novo*	18.53	-	LP	PS2 PM1 PM2 PM4	not present
16	Spl	c.1171+2_1171+4del	?	g.11783850_11783852del	hem	9	*de novo*	-	-	P	PVS1 PS2 PM2	not present
17	NS	c.961C>T	p.(Gln321*)	g.11783638C>T	hem	9	*de novo*	39	-	P	PVS1 PS2 PM2 PP3	not present
18	MS	c.1373G>T	p.(Arg458Leu)	g.11790366G>T	hem	11	*de novo*	23.4	4.687	LP	PS2 PM1 PM2 PP3 BP1	not present
19	NS	c.1314C>A	p.(Tyr438*)	g.11790307C>A	hem	11	*de novo*	32	-	P	PVS1 PS2 PM2 PP3	not present
20	NS	c.590_593del	p.(Leu197*)	g.11780957_11780960del	hem	7	unknown	32	-	P	PVS1 PS1 PM2 PP3	not present
21	NS	c.865A>T	p.(Lys289*)	g.11782014A>T	hem	8	*de novo*	37	-	P	PVS1 PS2 PM2 PP3	not present
22	NS	c.1347C>A	p.(Tyr449*)	g.11790340C>A	hem	11	*de novo*	33	-	P	PVS1 PS2 PM2 PP3	not present
23	NS	c.566dup	p.(Tyr189*)	g.11780349dup	het	6	*de novo*	32	-	P	PVS1 PS2 PM2 PP3	not present
24	FS	c.973_974del	p.(Gln326Alafs*5)	g.11783650_11783651del	hem	9	*de novo*	33	-	P	PVS1 PS2 PM2 PP3	not present
25	NS	c.1372C>T	p.(Arg458*)	g.11790365C>T	het	11	*de novo*	35	-	P	PVS1 PS2 PM2 PP3	not present

	Abbreviations: Spl = splicing, FS = frameshift, NS = nonsense, MS = missense, InDel = In-Frame-Deletion, het = heterozygous, hem = hemizygous, P = pathogenic, LP = likely pathogenic,

Table S3: Number of all newly (this study) and previously variants in *MSL3* (Basilicata et al[10], Clinvar[11]). Deletions spanning more genes than *MSL3* were excluded.

Exon number	Nucleotide alteration (NM_078629.4)	Protein alteration (NP_523353.2)	Genomic position hg19 (NC_000023.10)	Type of variant	Published record	Individual/Variation ID
6	c.566_567del	p.(Tyr189Leufs*3)	g.11780349_11780350del	FS	PMID: 30224647	P7
6	c.566dup	p.(Tyr189*)	g.11780349dup	NS	This study	23
6-8	c.(465+1_466-1)_(908+1_909-1)del	?	g.(11779702_11780248)_(11782058_11783585)del	Multi-Exon Deletion	this study	7
7	c.589-4_591del	?	g.11780952_11780958del	Spl	this study	4
7	c.590_593del	p.(Leu197*)	g.11780957_11780960del	NS	this study	14
7	c.590_593del	p.(Leu197*)	g.11780957_11780960del	NS	this study	20
8	c.808_809del	p.(Pro270Valfs*8)	g.11781957_11781958del	FS	this study	6
8	c.841C>T	p.(Gln281*)	g.11781990C>T	NS	PMID: 30224647	P13
8	c.865A>T	p.(Lys289*)	g.11782014A>T	NS	this study	21
9	c.913C>T	p.(Gln305*)	g.11783590C>T	NS	this study	9
9	c.938dup	p.(Leu314Phefs*18)	g.11783615dup	FS	PMID: 30224647	P9
9	c.961C>T	p.(Gln321*)	g.11783638C>T	NS	this study	17
9	c.973_974del	p.(Gln326Alafs*5)	g.11783650_11783651del	FS	this study	24
9	c.1018del	p.(Ala340Leufs*9)	g.11783695delG	FS	PMID: 30224647	P8
9	c.1036C>T	p.(Gln346*)	g.11783713C>T	NS	PMID: 30224647	P3
9	c.1065_1066del	p.(Ala356Glnfs*3)	g.11783742_11783743del	FS	ClinVar	487569
9	c.1105C>T	p.(Gln369*)	g.11783782C>T	NS	this study	10
9	c.1125_1141dup17	p.(Met381Argfs*30)	g.11783802_11783818dup17	FS	this study	12

9	c.1125_1141dup17	p.(Met381Argfs*30)	g.11783802_11783818dup17	FS	PMID: 30224647	P10
9	c.1146delC	p.(Lys383Serfs*22)	g.11783823del	FS	this study	2
9	c.1168_1169del	p.(Lys390Glufs*6)	g.11783845_11783846del	FS	this study	8
9	c.1171+2_1171+4del	?	g.11783850_11783852del	Spl	this study	16
9	c.1171+2T>G	?	g.11783850T>G	Spl	PMID: 30224647	P11
10	Deletion Exon 10	?	?	Multi-Exon Deletion	PMID: 30224647	P12
10	c.1193C>A	p.(Ser398*)	g.11786713C>A	NS	Clinvar	504431
10	c.1208del	p.(Pro403Leufs*2)	g.11786728del	FS	ClinVar	520841
11	c.1319dup	p.(Gly441Argfs*2)	g.11790312dup	FS	this study	5
11	c.1310A>C	p.(Asn437Thr)	g.11790303A>C	MS	this study	3
11	c.1314C>A	p.(Tyr438*)	g.11790307C>A	NS	this study, Zech et al.[7]	19
11	c.1347C>A	p.(Tyr449*)	g.11790340C>A	NS	this study	22
11	c.1362_1364del	p.(Gln454del)	g.11790355_11790357del	InDel	this study	15
11	c.1370T>C	p.(Leu457Pro)	g.11790363T>C	MS	this study and PMID:30224647 (referred to as c.923T> C, p.(Leu308Pro))	13, P4
11	c.1372C>T	p.(Arg458*)	g.11790365C>T	NS	PMID: 30224647	P5, P6
11	c.1372C>T	p.(Arg458*)	g.11790365C>T	NS	this study	25
11	c.1373G>T	p.(Arg458Leu)	g.11790366G>T	MS	this study	18
11	c.1374_1381del	p.(Leu459Glufs*13)	g.11790367_11790374del	FS	PMID: 30224647	P2
11	c.1381+1G>T	?	g.11790375G>T	Spl	PMID: 30224647	P1
12	c.1382-1G>A	p.?	g.11790739G>A	Spl	this study	11
12	c.1436dup	p.(Leu480Phefs*6)	g.11790794dup	FS	ClinVar	487571
12	c.1466+1G>A	p.?	g.11790825G>A	Spl	this study	1
13	c.1516_1517delinsA	p.(Ala506Metfs*23)	g.11793148_11793149delinsA	FS	ClinVar	487570

Abbreviations: Spl = splicing, FS = frameshift, NS = nonsense, MS = missense, InDel = In-Frame-Deletion

Table S4: Comparative analyses of the clinical features between females and males; and between individuals with loss-of-function variants and individuals with missense variants/in-frame deletions in *MSL3* (Fisher's exact text[12]).

Clinical features	Number	Female	Males	p-value	Number	Loss-of-Function-Variant	Missense-Variant/In-Frame-Deletion	p-value
Developmental delay (DD)	25	10	15	-	25	21	4	-
+	25	10	15		25	21	4	
-	0	0	0		0	0	0	
Intellectual Disability (ID)	16	7	9	0.4375	16	14	2	1
+	15	6	9		15	13	2	
-	1	1	0		1	1	0	
Autism-spectrum disorder (ASD)	20	9	11	1	20	16	4	0.582
+	10	4	6		10	9	1	
-	10	5	5		10	7	3	
Macrocephaly (including relative macrocephay)	21	8	13	0.0805	21	17	4	0.5865
+	11	2	9		11	8	3	
-	10	6	4		10	9	1	
Hypotonia	25	10	15	1	25	21	4	0.5269
+	21	8	13		21	18	3	
-	4	2	2		4	3	1	
Spasticity	23	10	13	0.65	23	19	4	0.5573
+	7	4	3		7	5	2	
-	16	6	10		16	14	2	
Brain MRI abnormalities	23	9	14	0.657	23	19	4	1
+	15	5	10		15	12	3	
-	8	4	4		8	7	1	
Seizures	23	9	14	1	23	20	3	0.4529
+	4	2	2		4	3	1	
-	19	7	12		19	17	2	
Dystonia	25	10	15	0.3577	25	21	4	1
+	5	3	2		5	4	1	
-	20	7	13		20	17	3	

Ataxia	23	10	13	1	23	20	3	0.5392
+	6	3	3		6	6	0	
-	17	7	10		17	14	3	
Brady-/hypokinesia	24	10	14	0.1921	24	21	3	0.5464
+	6	4	2		6	6	0	
-	18	6	12		18	15	3	
Skeletal features	24	10	14	1	24	20	4	1
+	17	7	10		17	14	3	
-	7	3	4		7	6	1	
Hearing abnormalities	23	10	13	0.685	23	19	4	0.5889
+	8	4	4		8	6	2	
-	15	6	9		15	13	2	
Disorder of the visual system	22	10	12	0.3913	22	19	3	1
+	12	4	8		12	10	2	
-	10	6	4		10	9	1	
Gastrointestinal disorder	23	10	13	0.1269	23	20	3	1
+	18	6	12		18	15	3	
-	5	4	1		5	5	0	
Urinary symptoms	23	9	14	0.6106	23	19	4	1
+	5	1	4		5	4	1	
-	18	8	10		18	15	3	
Respiratory symptoms	21	9	12	1	21	18	3	0.2571
+	8	3	5		8	8	0	
-	13	6	7		13	10	3	
Abnormalities of the cardiovascular system	23	10	13	0.6036	23	19	4	1
+	4	1	3		4	3	1	
-	19	9	10		19	16	3	
Loss-of-Function-Variant	25	10	15	0.6265	-	-	-	-
+	21	9	12		-	-	-	
-	4	1	3		-	-	-	

References

1. Li H, Durbin R. Fast and accurate short read alignment with Burrows-Wheeler transform. Bioinformatics. 2009;25(14):1754-1760.
2. Ye K, Schulz MH, Long Q, Apweiler R, Ning Z. Pindel: a pattern growth approach to detect break points of large deletions and medium sized insertions from paired-end short reads. Bioinformatics. 2009;25(21):2865-2871.
3. Plagnol V, Curtis J, Epstein M, et al. A robust model for read count data in exome sequencing experiments and implications for copy number variant calling. Bioinformatics. 2012;28(21):2747-2754.
4. Pezzani L, Marchetti D, Cereda A, et al. Atypical presentation of pediatric BRAF RASopathy with acute encephalopathy. Am J Med Genet A. 2018;176(12):2867-2871.
5. Retterer K, Juusola J, Cho MT, et al. Clinical application of whole-exome sequencing across clinical indications. Genet Med. 2016;18(7):696-704.
6. Wei W, Tuna S, Keogh MJ, et al. Germline selection shapes human mitochondrial DNA diversity. Science. 2019;364(6442).
7. Zech M, Jech R, Boesch S, et al. Monogenic variants in dystonia: an exome wide sequencing study. Lancet Neurol. 2020, in Press
8. Rentzsch P, Witten D, Cooper GM, Shendure J, Kircher M. CADD: predicting the deleteriousness of variants throughout the human genome. Nucleic Acids Res. 2019;47(D1):D886-D894.
9. Pollard KS, Hubisz MJ, Rosenbloom KR, Siepel A. Detection of nonneutral substitution rates on mammalian phylogenies. Genome Res. 2010;20(1):110-121.
10. Basilicata MF, Bruel AL, Semplicio G, et al. De novo mutations in MSL3 cause an X-linked syndrome marked by impaired histone H4 lysine 16 acetylation. Nat Genet. 2018;50(10):1442-1451.
11. Landrum MJ, Lee JM, Benson M, et al. ClinVar: improving access to variant interpretations and supporting evidence. Nucleic Acids Res. 2018;46(D1):D1062-D1067.
12. Fisher RA. On the interpretation of χ 2 from contingency tables, and the calculation of P. Journal of the Royal Statistical Society. 1922;85(1):87-94.
